# Surgery after induced anti-PD-L1 therapy and chemotherapy for stage I**‒**III small-cell lung cancer: a phase 2 trial (LungMate-005)

**DOI:** 10.1038/s41421-025-00838-5

**Published:** 2025-11-25

**Authors:** Fenghuan Sun, Lele Zhang, Liangdong Sun, Di Wang, Nan Song, Liang Duan, Dongliang Bian, Junjie Hu, Yilv Yan, Jie Yang, Wenxin He, Yong Yang, Xiaogang Liu, Bin Chen, Jun Ma, Lixin Wang, Ming Liu, Xiaoxiong Xu, Cong Ye, Yirui Zhou, Huansha Yu, ZhaoXia Dai, Chang Chen, Deping Zhao, Jie Luo, Shuyan Meng, Gening Jiang, Peng Zhang

**Affiliations:** 1 Department of Thoracic Surgery, Shanghai Pulmonary Hospital, School of Medicine, Tongji University, Shanghai, China; 2 Central Laboratory, Innovation and Incubation Center, Shanghai Pulmonary Hospital, School of Medicine, Tongji University, Shanghai, China; 3 Tissue Bank, Innovation and Incubation Center, Shanghai Pulmonary Hospital, School of Medicine, Tongji University, Shanghai, China; 4 Department of Oncology, Shanghai Pulmonary Hospital, School of Medicine, Tongji University, Shanghai, China; 5 Department of Radiology, Shanghai Pulmonary Hospital, School of Medicine, Tongji University, Shanghai, China; 6 Department of Traditional Chinese Medicine, Shanghai Pulmonary Hospital, School of Medicine, Tongji University, Shanghai, China; 7 Experimental Animal Center, Innovation and Incubation Center, Shanghai Pulmonary Hospital, Tongji University School of Medicine, Shanghai, China; 8 Department of Thoracic Medical Oncology II, The Second Hospital of Dalian Medical University, Dalian, Liaoning China

**Keywords:** Small-cell lung cancer, Tumour immunology

## Abstract

Immunochemotherapy has shown promising outcomes in treating small-cell lung cancer. To explore whether surgery after immunochemotherapy benefits patients with stage I‒III small-cell lung cancer, we conducted a phase II trial (NCT04539977). Eligible patients received four cycles of anti-PD-L1 antibody (TQB2450) therapy and chemotherapy, followed by surgery or radiotherapy and one-year maintenance immunotherapy (TQB2450). Forty patients were enrolled between December 2020 and January 2023. Thirty-eight (95.0%) patients had stage III disease. We found that the objective response rate, as the primary endpoint of this study, was 92.5% (95% CI: 83.9%‒100%) in the intention-to-treat population. At a median follow-up of 25.8 months, the median event-free survival (EFS) was 16.2 months. The median overall survival (OS) was not reached. The major pathological response and pathological complete response rate of operative patients (*n* = 21) were 61.9% and 42.9%, respectively. The 24-month EFS and 24-month OS of operative patients were 61.9% and 85.7%, respectively. All patients with N1 disease (*n* = 9) underwent surgery, with the 24-month EFS of 66.7% and 24-month OS of 88.9%. The most common TQB2450-specific adverse event was rash of grade 1‒2 (12.5%). We further explored the biomarker of immunochemotherapy and molecular changes during immunochemotherapy through bulk-RNA sequencing and whole-exome sequencing. We demonstrated that *PRSS8* was a potential biomarker for poor effectiveness of immunochemotherapy. In conclusion, surgery after neoadjuvant immunochemotherapy is feasible for treating patients with stage I‒III small-cell lung cancer.

## Introduction

Stage I‒III small-cell lung cancer (SCLC) is characterized by poor prognosis with a median progression-free survival (PFS) of 14‒20 months and an overall survival (OS) of 16‒30 months after systematic therapy^1^. According to National Comprehensive Cancer Network (NCCN) guideline, the standard treatment for stage I‒IIa and IIb‒III SCLC is surgery and chemoradiotherapy, respectively^2^. A prospective trial reported by Chest in 1994 showed that the median OS of patients with stage I‒III SCLC who received surgery after chemotherapy was 15.4 months, which was statistically equivalent to that after chemoradiotherapy (18.6 months) (*P* = 0.78)^3^. Therefore, surgery was not recommended for these patients considering the trauma and complications it brings. Although some studies in the following decades have demonstrated that surgery may provide survival benefits over radiotherapy^4–6^, guidelines have not changed because of insufficient evidence from retrospective studies.

Immunotherapy prolongs the survival of patients with stage I‒III SCLC in some trials^7–9^. Pembrolizumab or durvalumab combined with concurrent chemoradiotherapy (CCRT) yielded favourable outcomes in treating stage I‒III SCLC, with a median PFS of 19.6 or 14.4 months, respectively^7,8^. Durvalumab therapy after standard concurrent platinum-based chemoradiotherapy led to significantly longer OS than placebo (median OS: 55.9 vs 33.4 months), as well as to longer PFS (median PFS: 16.6 vs 9.2 months) in ADRIATIC trial^9^.

With the development of surgical technology and immunotherapy, whether surgery could benefit patients with stage I‒III SCLC after immunochemotherapy remains elusive. To explore the safety and effectiveness of surgery after treatment with induced anti-PD-L1 antibody (TQB2450) therapy and chemotherapy in patients with stage I‒III SCLC, we performed this phase II trial (LungMate-005).

Effective biomarkers for predicting the sensitivity of immunochemotherapy in treating SCLC are an urgent need. Recently, “SCLC-I” subtype, which is characterized by low expression of ASCL1, NEUROD1, and POU2F3 accompanied by an inflammatory gene signature, is considered to obtain the greatest benefit from immunochemotherapy^10,11^. Moreover, the impact of immunochemotherapy on the tumour microenvironment (TME) of SCLC remains poorly understood. Thus, we conducted bulk RNA sequencing and whole-exome sequencing (WES) to identify patients with stage I‒III SCLC who could benefit from induced immunochemotherapy.

The primary aim of this study was to explore whether surgery after treatment with induced anti-PD-L1 antibody (TQB2450) therapy and chemotherapy could be a beneficial option for patients with stage I‒III SCLC. The exploratory outcomes were biomarkers that could predict the effectiveness of immunochemotherapy and molecular changes in the TME after induced immunochemotherapy.

## Results

### Patient characteristics and treatment

**Fig. 1:**
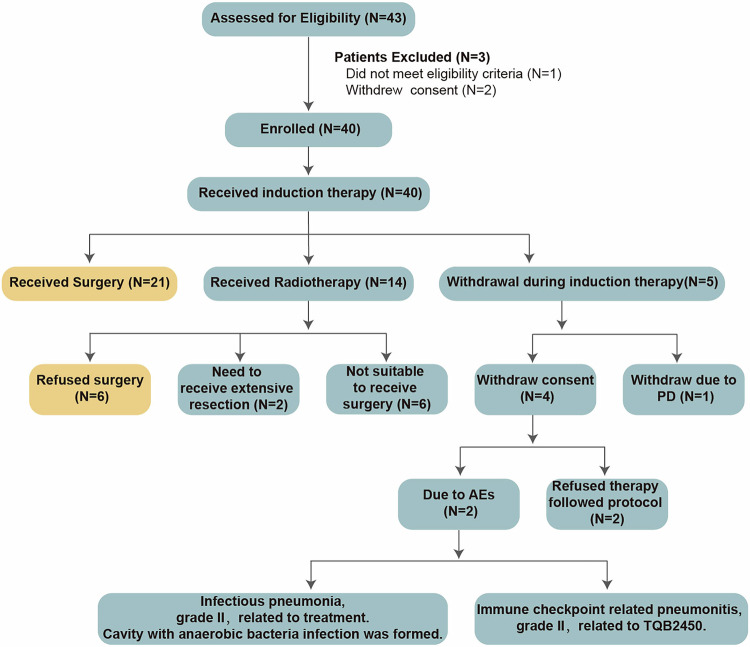
Patient flow diagram for LungMate-005. The flow diagram for patients screening, enrolment, receiving immunochemotherapy, receiving surgery (yellow) or radiotherapy, withdrawing consent, and exiting. Reasons for the need to receive extensive resection: (1) Patient 1: pneumectomy was needed due to a tumour adjacent to the left main pulmonary artery. (2) Patient 2: the tumour was located across the pulmonary fissure and invaded both the right upper lobe and right lower lobe. Reasons for not receiving surgery: (1) A patient who suffered from pulmonary arterial hypertension. (2) A patient who had a migratory pulmonary infection or a highly suspected fungal infection. (3) The patient had bulky lymph node (N2) involvement. The diameter of group 4 lymph node was largely decreased after four cycles of induction therapy. But the group 4 lymph node was still pathology confirmed positive after four cycles of induction therapy. (4) The tumour subsided but cancerous lymphangitis developed in one patient after induction therapy. (5) The metastatic lymph nodes of the two patients surrounded blood vessels, which were difficult to separate and inoperable.

Between December 2020 and January 2023, 43 patients were screened, 40 of whom were enrolled (Fig. 1). Thirty-eight (95.0%) patients had stage III disease. The demographics and baseline characteristics are given in Table 1. Among 40 eligible patients, twenty-one patients received surgery after induction therapy, and fourteen patients received radiotherapy (those who were refused or unfit for surgery; the detailed reasons are shown in Fig. 1). The other five patients withdrew from the trial during induction therapy (Detailed reasons are shown in Fig. 1). The median age of patients who received surgery after immunochemotherapy was 58 years (IQR 52, 65). Twenty (95.2%) patients were at clinical stage III. Nine (42.9%), seven (33.3%), and four (19.0%) patients were diagnosed with clinical N1, N2, and N3 disease, respectively (Table 1).

**Table 1:** Demographics and baseline characteristics.

Characteristic		Total (*n* = 40)	Surgery (*n* = 21)	Radiotherapy (*n* = 14)	Withdrew^a^ (*n* = 5)
*n* (%) with gender	male	35 (87.5)	19 (90.5)	12 (85.7)	4 (80.0)
Age, median (IQR)		61 (56, 69)	58 (52,65)	69 (59,72)	64 (57,68)
*n* (%) with smoking status	ever-smoker	28 (70.0)	15 (71.4)	10 (71.4)	3 (60.0)
	never-smoker	12 (30.0)	6 (28.6)	4 (28.6)	2 (40.0)
*n* (%) with comorbidity	yes	22 (55.0)	10 (47.6)	10 (71.4)	2 (20.0)
*n* (%) with tumour site	right lung	22 (55.0)	11 (52.4)	7 (50.0)	4 (80.0)
	left lung	18 (45.0)	10 (47.6)	7 (50.0)	1 (20.0)
*n* (%) with T	1	3 (7.5)	2 (9.5)	1 (7.1)	0 (0.0)
	2	7 (17.5)	4 (19.0)	1 (7.1)	2 (40.0)
	3	7 (17.5)	2 (9.5)	4 (28.6)	1 (20.0)
	4	23 (57.5)	13 (61.9)	8 (57.1)	2 (40.0)
*n* (%) with *N*	0	2 (5.0)	1 (4.8)	1 (7.1)	0 (0.0)
	1	9 (22.5)	9 (42.9)	0 (0.0)	0 (0.0)
	2	21 (52.5)	7 (33.3)	9 (64.3)	5 (100.0)
	3	8 (20.0)	4 (19.0)	4 (28.6)	0 (0.0)
*n* (%) with clinical stage	IB	1 (2.5)	1 (4.8)	0 (0.0)	0 (0.0)
	IIB	1 (2.5)	0 (0.0)	1 (7.1)	0 (0.0)
	IIIA	16 (40.0)	12 (57.1)	2 (14.3)	2 (40.0)
	IIIB	16 (40.0)	6 (28.6)	7 (50.0)	3 (60.0)
	IIIC	6 (15.0)	2 (9.5)	4 (28.6)^b^	0 (0.0)
*n* (%) with site of neoplasm in tracheobronchial	no	19 (47.5)	11 (52.4)	5 (35.7)	3 (60.0)
	segmental bronchus	10 (25.0)	6 (28.6)	3 (21.4)	1 (20.0)
	lobar bronchus	9 (22.5)	4 (19.0)	4 (28.6)	1 (20.0)
	left main bronchus	2 (5.0)	0 (0.0)	2 (14.3)	0 (0.0)
*n* (%) with PD-L1 expression	0	22 (55.0)	9 (22.5)	10 (25.0)	3(7.5)
	$\ge$1%	9 (22.5)	6 (15.0)	2 (5.0)	1 (2.5)
	NA	9 (22.5)	6 (15.0)	2 (5.0)	1 (2.5)

^a^ Withdrew includes those who did not complete surgery or radiotherapy following the protocol.

^b^ One patient with superior vena cava syndrome was enrolled. His symptoms of superior vena cava compression eased significantly after interventional stent implantation surgery.

Three patients did not receive complete induction therapy according to the protocol, instead receiving only 1 cycle of induction therapy (Supplementary Fig. S1). Among patients who underwent surgery, four patients required surgery before the planned date of the protocol: three after receiving 2 cycles of treatment and one after receiving 3 cycles of treatment. Among the patients who received radiotherapy, two patients received 6 cycles of induction therapy due to the strong desire to receive surgery. Thirty-one patients (including two patients who did not receive surgery or radiotherapy according to the protocol after induction therapy) received four cycles of induction therapy.

### Safety of immunochemotherapy

The incidence of adverse events (AEs) overall and of grade 3–5 AEs were 100% and 47.5%, respectively. Treatment-related AEs with an incidence of at least 10% in any grade category included alopecia (40, 100.0%), decreased white blood cells (22, 55.0%), decreased platelet count (18, 45.0%), decreased neutrophil count (15, 37.5%), increased γ-glutamyl transferase (13, 32.5%), anaemia (12, 30.0%), increased alkaline phosphatase (10, 25.0%), increased alanine aminotransferase (8, 20.0%), rash (5, 12.5%), increased aspartate aminotransferase (5, 12.5%), and increased creatinine (5, 12.5%). Most treatment-related AEs were TQB2450 nonspecific. TQB2450-specific AEs included rash (5, 12.5%), hyperglycaemia (3, 7.5%), and thyroid dysfunction (3, 7.5%).

The incidence of severe adverse events (SAEs) of any grade and of grade 3‒5 was 17.5% (7 patients) and 12.5% (5 patients), respectively. Only one patient experienced immunochemotherapy-related SAEs. This patient was hospitalized with a grade 3 SAE of decreased platelet count. Two patients experienced disease-related SAEs: one was hospitalized with grade 3 hypotension, and the other was hospitalized with grade 2 hydrothorax caused by disease progression. Two patients developed grade 3 bronchopleural fistula related to surgery, and both were treated successfully with adequate drainage. One patient experienced grade 3 radiation pneumonitis and died due to grade 5 acute kidney injury. One patient had a grade 2 thrombus in the implantable venous access port, which was related to irregular access port nursing.

### Safety of surgery

**Fig. 2:**
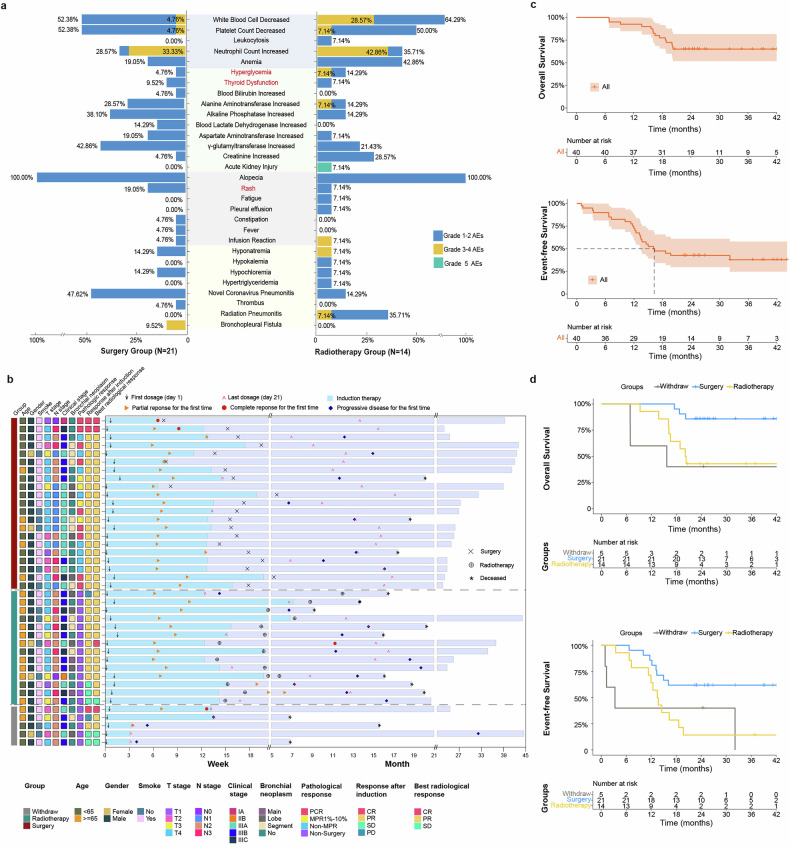
Safety and effectiveness of surgery or radiotherapy after treatment with anti-PD-L1 antibody (TQB2450) therapy and chemotherapy followed by maintenance therapy. **a** Incidence of AEs in the surgery group (*n* = 21) and radiotherapy group (*n* = 14). AEs marked in red are TQB2450-specific AEs. **b** The baseline characteristics and effectiveness. **c** EFS and OS of the intention-to-treat population. **d** EFS and OS of patients who received surgery (blue), radiotherapy (yellow), or withdrew (grey). Survival outcomes were analyzed at the time of data cut-off (September 30, 2024).

Following preoperative assessment, twenty-seven (27/40, 67.5%) patients were considered appropriate candidates for curative surgery after induced immunochemotherapy. Six of them refused surgery, the other 21 received surgery. Eighteen (18/21; 85.7%) patients underwent lobectomy, and the other 3 (3/21; 14.3%) patients underwent sleeve resection. The detailed surgical approaches used are listed in Table 2. The median intraoperative blood loss was 50 mL (IQR: 50, 50 mL). The 30-day and 90-day mortality rates were both 0%. Four (4/21; 19.0%) patients had surgical complications: two (2/21; 9.5%) patients experienced bronchopleural fistula, and two (2/21; 9.5%) patients experienced subcutaneous emphysema. The detailed distribution of AEs in patients who received surgery or radiotherapy is reported in Fig. 2a.

**Table 2:** Safety and effectiveness in the surgery group (*n* = 21).

Characteristics	Surgery (*n* = 21)
Time from last dose chemotherapy to surgery, days	Mean ± SD: 40.24 ± 11.19 IQR: 39.00 (32.00, 46.00)
R0 resection	100%
*n* (%) with surgical approaches,	
RATS lobectomy	2 (9.5%)
VATS lobectomy	11 (52.3%)
VATS bi-lobectomy	2 (9.5%)
VATS double sleeve resection (Bronchovascular)	1 (4.8%)
Lobectomy	2 (9.5%)
Tracheal sleeve resection	1 (4.8%)
Double sleeve resection (Bronchovascular)	1 (4.8%)
Intrapericardial bi-lobectomy	1 (4.8%)
Operation time, hours	Mean ± SD: 2.07 ± 0.62 IQR: 2.00 (1.50, 2.50)
Intraoperative blood loss, mL	Mean ± SD: 63.81 ± 42.01 IQR: 50.00 (50.00, 50.00)
Blood transfusion, mL	0 (no one received blood transfusion)
Hospitalization time, days	Mean ± SD: 5.38 ± 0.42 IQR: 5.00 (4.00, 7.00)
Chest tube removal time, days	Mean ± SD: 11.67 ± 5.62 IQR: 11.00 (6.50, 15.00)
30-days mortality	0
90-days mortality	0
*n* (%) with complications,	4 (19.0%)
Bronchopleural fistula	2 (9.5%)
Subcutaneous emphysema	2 (9.5%)
*n* (%) with residual viable tumour	
0%	9 (42.9%)
0–10%	4 (19.0%)
> 10%	8 (38.1%)
Residual viable tumour, %	Mean ± SD: 18 ± 27 IQR: 5.00 (0.00, 20.00)
Decrease in sum of lesion diameter, %	Mean ± SD: 59.84 ± 21.98 IQR: 62.48 (40.44, 78.78)
*n* (%) with TNM downstaging	17 (81.0%)
*n* (%) with T downstaging	19 (90.5%)
*n* (%) with N downstaging^a^	15 (71.4%)
N3 to N0	3 (75.0%)
N3 to N1	1 (25.0%)
N2 to N0	3 (42.9%)
N2 to N2	4 (57.2%)
N1 to N0	8 (88.9%)
N1 to N1	1 (11.1%)

^a^ The diameter of the lymph node (N2) was smaller than 15 mm. PET-CT indicated that lymph nodes (N2) were negative before surgery; lymph nodes (N3) were confirmed to be pathologically negative before surgery. No systematic dissection of the lymph nodes (N3) was performed. lymph nodes (N3) were pathologically confirmed negative by endobronchial ultrasound, supraclavicular lymph node puncture biopsy, or mediastinoscopy.

### Radiological response

In the intention-to-treat population, three patients achieved complete response (CR), thirty-four patients achieved partial response (PR), and three patients achieved stable disease after TQB2450 and chemotherapy (Fig. 2b). The objective response rate (ORR) was 92.5% (95% CI: 83.9%–100%). The ORR after 2 or 4 cycles of induction therapy was 80.0% (95% CI: 67.0%–93.0%) and 87.5% (95% CI: 76.8%‒98.2%), respectively. In most patients who achieved an objective response, the response was observed at the first planned evaluation, which was 6 weeks after the initiation of treatment (Fig. 2b). The median duration of overall response was 14.7 (95% CI: 7.4‒21.9) months in the intention-to-treat population. The median duration of overall response was 11.3 (95% CI: 8.9‒13.8) months in the radiotherapy group, but it was not reached in the surgery group.

### Pathological response in the surgery group

Twenty-one patients who underwent surgery achieved pathological response, including nine (9/21; 42.9%) patients who achieved pathological complete response (PCR), four (4/21; 19.0%) patients who achieved major pathological response 1%–10% (MPR 1%–10%), and eight (8/21; 38.1%) patients who did not achieve major pathological response (non-MPR). The MPR (0‒10%) rate was 61.9% (13/21) (Fig. 2b). The rates of MPR and PCR in the ITT population were 32.5% (13/40) and 22.5% (9/40), respectively. The surgical rates according to nodal status were 50% in N0 disease, 100% in N1 disease, 41.67% in N2 disease, and 50% in N3 disease, respectively. The MPR rates according to nodal status were N0 100%, N1 66.7%, N2 30%, and N3 75%, respectively. The PCR rate in those with node-negative and node-positive (N1‒N3) was 100%, 33.4%, 20%, and 75%, respectively. Seventeen (17/21; 81.0%) patients achieved pathological downstaging after surgery (Table 2). Detailed information about surgical outcomes is reported in Table 2.

### Survival outcomes

At the time of data cut-off (September 30, 2024), the median follow-up was 25.8 (95% CI: 23.5‒28.2) months. In the intention-to-treat population, twenty-four patients experienced disease progression. Among them, seven patients (7/24) had brain metastases, two (2/24) had liver metastases, one (1/24) had both brain and liver metastases, and the others (14/24) had enlarged pulmonary lymph nodes or primary tumours. The median event-free survival (EFS) was 16.2 (95% CI: 13.3-not reached) months. The 12-month EFS and 24-month EFS was 70.9% (95% CI: 59.9%‒87.7%) and 42.5% (95% CI: 29.6%‒60.9%), respectively (Fig. 2c).

Fourteen patients died. Ten of them died due to disease progression, two died due to COVID-19, one died of renal failure, and the other one died of diabetic ketoacidosis. The median overall survival (OS) was not reached. The 12-month OS and 24-month OS was 92.5% (95%: CI 84.7%‒100.0%) and 75.4% (95%: CI 51.8%‒81.6%), respectively (Fig. 2c).

In the surgery group, the median EFS and median OS were not reached. The 24-month EFS and 24-month OS were 61.9% (95% CI: 44.3%‒86.6%) and 85.7% (95% CI: 72.0%‒100.0%), respectively (Fig. 2d). The median disease-free survival in surgery group was not reached. The 24-month disease-free survival was 61.9% (95% CI: 44.3%‒86.6%). The 24-month EFS and 24-month OS of patients who achieved MPR were 76.9% (95% CI: 57.1%‒100%) and 84.6% (95% CI: 67.1%‒100%), respectively. None of those who achieved PCR experienced recurrence or death. The 24-month EFS of patients with N1, N2, and N3 positive disease was 66.7% (95% CI: 42.0%‒100%), 33.3% (95% CI: 18.2%‒61.0%), and 37.5% (95% CI: 15.3%‒91.7%), respectively. The 24-month OS of patients with N1, N2, and N3 positive disease was 88.9% (95% CI: 70.6%‒100%), 57.1% (95% CI: 39.5%‒82.8%), and 62.5% (95% CI: 36.5%‒100%), respectively.

In the radiotherapy group, the median EFS and median OS were 13.6 (95% CI: 12.0-not reached) and 20.2 (95% CI: 16.5-not reached) months, respectively. The 24-month EFS and 24-month OS were 14.3% (95% CI: 4.0%‒51.5%) and 42.9% (95% CI: 23.4%‒78.5%), respectively (Fig. 2d). The PFS of the radiotherapy group was the same as its EFS.

### Molecular differences

**Fig. 3:**
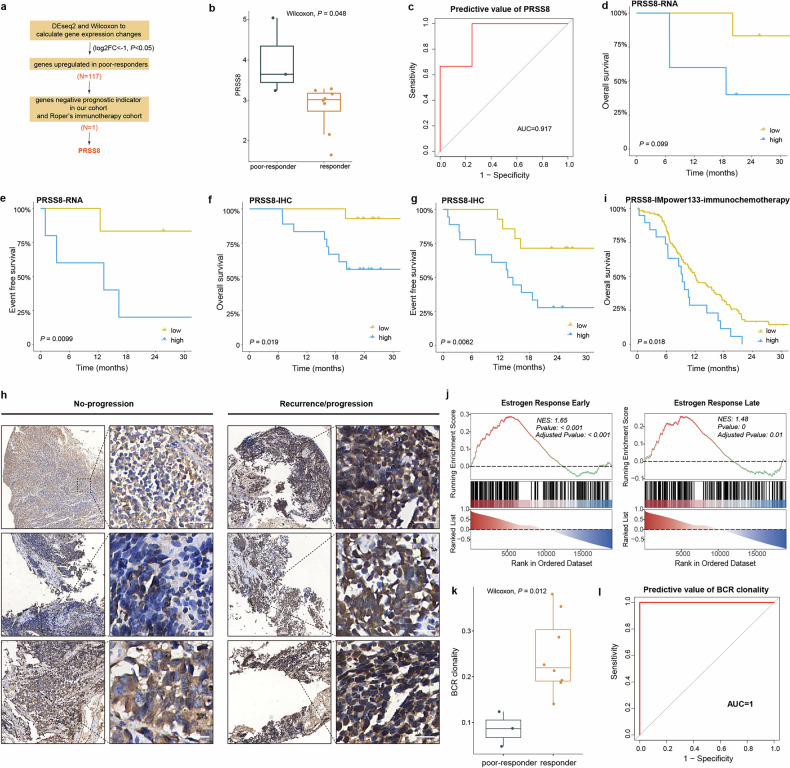
Transcriptomic features of patients with different responses. **a** Flow diagram for screening PRSS8 as predictor of effectiveness of immunotherapy. **b** Higher *PRSS8* mRNA expression (Log_2_ (CPM + 1)) in poor-responder tumours. **c** *PRSS8* mRNA expression in pretreatment samples predicted the effectiveness of immunochemotherapy. **d**, **e** Differences in OS (**d**) and EFS (**e**) between patients with high and low *PRSS8* mRNA expression in the discovery cohort (*n* = 11). **f**, **g** Differences in OS (**f**) and EFS (**g**) between patients with high and low PRSS8 IHC expression in our cohort (*n* = 34). **h** Representative IHC images (5× and 40×) of PRSS8. The tissue sections were visualized by microscopy. Scale bars represent 50 µm. **i** The difference in OS between patients who received immunochemotherapy with high and low *PRSS8* mRNA expression in IMpower133 cohort (*n* = 132). **j** Significant enrichment of the “estrogen response early” and “estrogen response late” pathways in patients with high *PRSS8* mRNA expression. **k**, **l** High BCR clonality in responder tumours (**k**) and discrimination performance for response of immunochemotherapy (**l**).

To identify predictors of the effectiveness of immunochemotherapy, differential gene expression analysis of baseline tumour samples from radiological responders (patients who achieved PR or CR, *n* = 8) and poor-responders (patients who achieved SD, *n* = 3) was performed (Supplementary Fig. S2a). A total of 3147 genes were differentially expressed: 390 genes were more highly expressed in responders, and 2757 genes were more highly expressed in poor-responders. Notably, *PRSS8* was significantly overexpressed in tumour samples from poor-responders (Fig. 3a, b). Besides, *PRSS8* was related to a worse radiological response in RNA-seq (AUC = 0.92, 95% CI: 0.72‒1; ACC = 0.82, 95%CI: 0.79‒0.85) (Fig. 3c). Furthermore, high *PRSS8* mRNA expression was associated with poor OS (*P* = 0.099) and EFS (*P* < 0.01) (Fig. 3d, e). SCLC with higher pretreatment PRSS8 expression, as determined by immunohistochemical (IHC) (*n* = 34), was also associated with worse OS (*P* = 0.02) and EFS (*P* = 0.01) (Fig. 3f, g). Representative immunohistochemical images of PRSS8 are shown in Fig. 3h. In another cohort of patients with SCLC receiving anti-PD-1/PD-L1 therapy, higher *PRSS8* expression was also associated with worse OS (*P* = 0.09)^12^ (Supplementary Fig. S2b). Furthermore, high mRNA expression of *PRSS8* was associated with poor OS (*P* = 0.02) (Fig. 3i) and PFS (*P* = 0.02) (Supplementary Fig. S2c) in IMpower133 cohort of patients with SCLC receiving immunochemotherapy (*n* = 132)^13^. Patients with higher expression of *PRSS8* mRNA showed enrichment of genes involved in estrogen response pathways (Fig. 3j), which might contribute to the worse therapeutic effect of immunochemotherapy^14^. Immune repertoire analysis revealed that B-cell receptor clonality was significantly higher in tumour samples from responders than in those from poor-responders (Fig. 3k), which also indicated good discrimination performance for the radiological response to immunochemotherapy in patients with stage I‒III SCLC (AUC = 1, 95% CI: 1‒1; ACC = 0.73, 95%CI: 0.69‒0.76) (Fig. 3l).

### Differential changes after induction therapy

We conducted WES on 17 samples (12 pretreatment and 5 posttreatment) (Supplementary Fig. S2a). The most frequent mutations occurred in *TP53*, *RB1*, etc., which was consistent with previous study^15,16^ (Supplementary Fig. S3a). The tumour mutation burden significantly decreased after immunochemotherapy (Supplementary Fig. S3b, c).

**Fig. 4:**
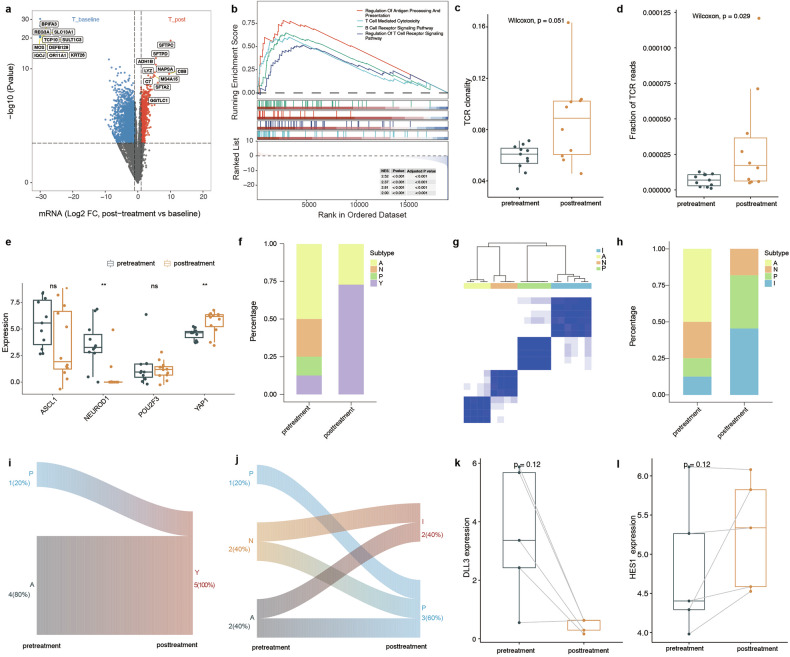
Dynamic changes within the tumour during induction therapy. **a** Differential expression between pretreatment (*n* = 11) and posttreatment (*n* = 12) tumour samples. **b** Significant enrichment of multiple immune pathways in posttreatment tumour samples. **c** Increased TCR clonality in posttreatment tumour samples. **d** Increasing fraction of TCR reads in posttreatment tumour samples. **e** The expression differences among *ASCL1*, *NEUROD1*, *POU2F3*, and *YAP1* between pretreatment and posttreatment tumour samples. **f** Increasing fractions of the Y subtype in posttreatment tumour samples. **g** Clustering for the ANPI subtypes in tumour samples. **h** Increasing fractions of the I subtype in posttreatment tumour samples. **i** ANPY subtype and induction therapy in patients with matched longitudinal samples. **j** ANPI subtype and induction therapy in patients with matched longitudinal samples. **k** Low *DLL3* expression after treatment in patients with matched longitudinal samples. **l** High *HES1* expression after treatment in patients with matched longitudinal samples. The units for the quantified *ASCL1, NEUROD1, POU2F3, YAP1, DLL3* and *HES1* expression are Log_2_ (CPM + 1).

Considering the potential differences in tumour microenvironment between primary tumours and metastatic lymph nodes^17,18^, we performed independent analyses for primary tumours and metastatic lymph node. Comparing pretreatment tumour samples (*n* = 11) to posttreatment tumour samples (*n* = 12) (Supplementary Fig. S2a), 3679 genes were differentially expressed (1180 genes were more highly expressed in posttreatment tumour samples, and 2499 genes were more highly expressed in pretreatment tumour samples) (Fig. 4a). Gene set enrichment analysis revealed that the “T cell-mediated cytotoxicity”, “regulation of T cell receptor signalling”, “B cell receptor signalling” and “regulation of antigen processing and presentation” pathways were enriched specifically in posttreatment tumour samples (Fig. 4b). T-cell receptor clonality and the fraction of T-cell receptor reads also increased in posttreatment tumour samples (Fig. 4c, d). In the comparison between pretreatment (*n* = 6) and posttreatment (*n* = 9) lymph node samples (Supplementary Fig. S2a), multiple immune checkpoint genes (Supplementary Fig. S4a) and some T-cell effector genes showed increasing trends in posttreatment lymph node samples (Supplementary Fig. S4b). The potential infiltration of multiple immune cells, such as B cells and monocytic lineage cells, was higher in posttreatment lymph node samples (Supplementary Fig. S4c). Along with high B-cell infiltration, high expression of MHC-II modules was observed in posttreatment lymph node samples (Supplementary Fig. S4d).

### ANPY/ANPI subtype change occurs during induction therapy

Considering the important roles of *ASCL1*, *NEUROD1*, *POU2F3*, and *YAP1* in SCLC, we evaluated the expression of these four master transcription factors (TFs). After treatment, *YAP1* expression increases while *ASCL1* and *NEUROD1* expressions decrease (Fig. 4e; Supplementary Fig. S5a‒d). The results of IHC staining also revealed decreased expression of NEUROD1 (Supplementary Fig. S5e, f) and increased expression of YAP1 (Supplementary Fig. S5g, h) after treatment. Representative images were presented in Supplementary Fig. S5i‒k. Notably, the percentage of the samples that enriched of *YAP1* was increasing in posttreatment tumour samples than pretreatment (*P* = 0.04, chi-squared test) (Fig. 4f). When we evaluated the association between the expression profile and the ANPI subtype (Fig. 4g), a high SCLC-I subtype percentage was observed in the posttreatment tumour group (*P* = 0.04, chi-squared test) (Fig. 4h).

In patients with matched longitudinal tumour samples, all patients might shift from the “A” or “P” to the “Y” subtype during treatment according to the ANPY subtype assessment (Fig. 4i). As for the ANPI subtype assessment, four patients with pretreatment subtype A or N shifted to subtype P or I, and one remained in subtype P after treatment (Fig. 4j). These results suggested a neuroendocrine state transition after induction therapy.

Previous studies have proposed that Notch activation is capable of mediating a switch between neuroendocrine and non-neuroendocrine SCLC cell fates^19^. We compared the expression of key upstream and downstream molecules in the *Notch* pathway between the baseline and posttreatment periods. We observed some evidence of increased *MYC* (an upstream molecule of Notch) (Supplementary Fig. S6a), decreased *DLL3* (the Notch inhibitory ligand) (Fig. 4k) and increased *HES1* (the Notch pathway transcriptional target) (Fig. 4l) expression in RNA-seq after treatment, but the differences were not statistically significant. IHC staining for DLL3 and HES1 also revealed some evidence of decreased expression of DLL3 (Supplementary Fig. S6b‒d) and slightly increased HES1 (Supplementary Fig. S6e) after treatment. These findings might suggest a potential role of MYC in activating NOTCH signalling to induce SCLC neuroendocrine state transition after immunochemotherapy^19,20^.

## Discussion

Surgery and chemoradiotherapy was recommended as the standard treatment for stage I‒IIa SCLC and stage IIb‒III SCLC in guidelines, respectively. With the development of surgery techniques and immunotherapy^9^, we wondered whether surgery could benefit and be a choice for patients with stage I‒III SCLC. In this study, we demonstrated that induced anti-PD-L1 antibody (TQB2450) therapy and chemotherapy was safe. In the intention-to-treat population, the incidence of grade 3‒5 AEs was 47.5%, and rash of grade 1‒2 was the most common TQB2450-specific AE (12.5%). Although eligible patients in this trial presented with advanced-stage disease (95.0% of them had stage III disease), the effectiveness of treatment was promising. In the intention-to-treat population, the ORR was 92.5%, the median EFS was 16.2 (95% CI: 13.3-not reached) months, and the 24-month OS was 75.4% (95% CI: 51.8%‒81.6%). Twenty-seven (67.5%) patients were considered appropriate candidates for curative surgery after induced immunochemotherapy. Especially, patients who received surgery achieved encouraging outcomes with the 24-month EFS of 61.9% (95% CI: 44.3%‒86.6%) and 24-month OS of 85.7% (95% CI: 72.0%‒100.0%), respectively (*n* = 21). The rates of MPR and PCR were 61.9% and 42.9%, respectively. A subset of SCLC patients with higher lymph node staging may also benefit from neoadjuvant chemotherapy and immunotherapy. *PRSS8* was potential biomarkers for poor effectiveness of immunochemotherapy.

The safety profile in the intention-to-treat population was manageable, with an overall incidence of AEs of 100% and of grade 3‒4 AEs of 47.5%, which was similar to earlier findings^1,21^. The incidence of any-grade AEs and grade 3‒4 AEs in patients with stage I‒III SCLC who received chemoradiotherapy and sequential PD-1 inhibitor maintenance therapy was 86.7%‒98.7% and 25.3%‒61.5%, respectively^1,21^. The most common grade 3‒4 treatment-related AEs in this study were haematological toxicities, with a frequency similar to that in a previous study. The most common AE in this study was grade 1‒2 alopecia (100%, related to chemotherapy), which was more common (33.7%‒44.0%) in ES-SCLC patients who received immunochemotherapy^22–25^. Approximately 35.7% of patients who received radiotherapy suffered from radiation pneumonitis, which is higher than the 21% incidence after concurrent chemoradiotherapy in the CONVERT study and the 16% incidence after pembrolizumab and concurrent chemoradiotherapy, but lower than the 52% after durvalumab and CCRT^1,7^. Thus, induced TQB2450 therapy and chemotherapy followed by surgery or radiotherapy were manageable considering the incidence of AEs.

CCRT is considered the standard treatment for stage I‒III SCLC, with an ORR ranging from 66%‒70%^1,7,8,26^. Only two trials have explored the effectiveness of CCRT and anti-PD-1/PD-L1 therapy and demonstrated that the ORR after CCRT with pembrolizumab or durvalumab was 79% and 96%, respectively^7,8^. The ORR of CCRT combined with anti-PD-1/PD-L1 therapy (79%‒96%) seems favourable compared to that of CCRT alone (66%‒70%)^1,7,8,26^. The ORR in another trial that enrolled 17 patients to assess the effectiveness of atezolizumab and chemotherapy in patients with stage I‒III SCLC was 94%^27^. Another retrospective study which explored the effect of immunochemotherapy followed by surgery reported an ORR of 84.2% (16/19)^28^. The ORR, as the main endpoint in this trial, was 92.5%, which seemed equivalent to that of CCRT combined with anti-PD-1/PD-L1 therapy in other trials and that of immunochemotherapy combined with surgery (79%‒96%)^1,7,8,26^. In summary, considering the ORR of this study, surgery or radiotherapy after induced anti-PD-L1 antibody (TQB2450) therapy and chemotherapy is feasible.

Considering surgery for stage III SCLC was not recommended in previous guidelines^2^, data on the surgical rate after induction therapy were lacking in previous studies. The surgical rate in previous trials of neoadjuvant immunochemotherapy treating stage IB**‒**IIIB non-small-cell lung cancer was 60%‒100%^29–33^. The surgical rate was slightly lower in the neoadjuvant chemotherapy group than in the neoadjuvant immunochemotherapy group^29–32,34^. A phase II trial revealed that the surgical rate after neoadjuvant SHR-1701 with or without chemotherapy among patients with unresectable pretreatment IIIA, IIIB, and IIIC non-small-cell lung cancer was 37% (10/27), 26% (12/47), and 16% (5/32), respectively^35^. In our trial, twenty-seven (67.5%) patients were considered appropriate candidates for curative surgery after induction therapy, and only 52.5% of patients underwent surgery. All the operative patients achieved R0 resection. In this trial, the surgical rate and R0 resection rate of patients with N1 disease were both 100%, which revealed that the choice of surgery after neoadjuvant chemotherapy and anti-PD-L1 antibody (TQB2450) therapy is feasible for N1 positive SCLC. Additional trials are still needed to explore the features of patients who was able to receive surgery after immunochemotherapy. Lymph nodes surrounding the main branches of the pulmonary artery and lymph node (N2) metastasis are the main reasons for inoperability. Thus, induced immunochemotherapy may improve survival in patients with stage I‒III SCLC. Patients who achieved partial responses but refused surgery mainly concerned about postoperative recovery and prognosis. Evidence-based confirmation of intraoperative and postoperative safety and effectiveness may increase surgical acceptance rates among those patients. More trials should be performed to explore the safety and effectiveness of surgery after neoadjuvant therapy in treating stage I‒III SCLC.

The rate of PCR after neoadjuvant chemotherapy in patients with stage I‒III SCLC was 19%^3^. Immunochemotherapy resulted in a PCR rate of 42.9% in our trial. In a trial including 11 patients and exploring the role of immunochemotherapy, the PCR was 61.5%^27^. The rate of pathological PCR and MPR was 30.0% (3/10), and 40.0% (4/10) in a retrospective study, respectively^28^. The differences in these results may be influenced by differences in the characteristics of enrolled patients and diagnostic technology. Limited by the diagnostic technology in 1994, patients who were not suitable for surgery after chemotherapy may also be enrolled, resulting in a low PCR rate in the trial which published in 1994. However, the PCR rate (42.9%) is still impressive. Thus, surgery after induced anti-PD-L1 antibody (TQB2450) therapy and chemotherapy could be an effective treatment for patients with stage I**‒**III SCLC from the perspective of pathological remission.

The survival outcomes of CCRT alone, CCRT combined with immunotherapy, and CCRT followed by immunotherapy were summarized in Table 3^1,8,9,21,26^. Considering the different distribution of stages of enrolled patients, those results cannot be compared directly. CCRT combined with anti-PD-1/PD-L1 therapy seems to have some advantages in improving overall survival compared to CCRT^1,8,26^. The survival of CCRT followed by immunotherapy also differs in two trials^9,21^. Considering more stage III patients enrolled in this trial, the median EFS (16.2 months in the intention-to-treat population) seems encouraging. Patients who received radiotherapy have advanced stages (50% patients of stage IIIB and 28.6% patients of stage IIIC), which may be related to the low PFS and OS. Although the 24-month PFS of patients in the radiotherapy group was 42.9%, it was also higher than that reported in the eighth-edition stage groups in the NCDB and IASLC data sets (19%‒32%)^36^. The 24-month PFS of patients of those who received sequential chemoradiotherapy and CCRT is 20% and 34.2%‒36.1%, respectively^9,26^. Thus, chemo-immunotherapy followed by radiotherapy and maintenance immunotherapy may serve as a viable alternative for patients who are unable to tolerant to CCRT. The survival outcome of patients who received surgery was especially encouraging with the 24-month EFS of 61.9% (95% CI: 44.3%‒86.6%) and 24-month OS of 85.7% (95% CI: 72.0%‒100.0%), respectively. Especially for patients with N1 disease, all of them received surgery with a 24-month EFS of 66.7% and 24-month OS of 88.9%, which revealed that this therapy could be a good choice considering its encouraging survival. Although this phase 2 trial was limited in its ability to evaluate the effectiveness by its small sample size and short follow-up, OS and EFS at the time of analysis were favorable. Favorable survival outcomes revealed that induced immunochemotherapy followed by surgery/radiotherapy and maintenance immunotherapy was feasible.

**Table 3:** Survival outcomes in trials of treating SCLC.

Treatment	Trials	*n*	Enrolled patients with stage III	24-month PFS	mPFS	24-month OS	mOS
CCRT	JCOG9104^26^	*n* = 114	57%IIIA+37%IIIB	30%	12	54.4%	27.2
	Convert (twice daily)^1^	*n* = 274	80%III	45%	15.4	56%	30
CCRT+ anti-PD-1/PD-L1 therapy	Pembroli-zumab with CCRT^7^	*n* = 40	83%III+2%IV	45%	19.7	65.8%	39.5
	Durvalumab with CCRT^8^	*n* = 50	54%IIIA+24%IIIB	42.0%	14.4	67.8%	NR^b^
CCRT followed by immuno-therapy	ETOP/IFCT 4-12 STIMULI^21^	Nivo-lumab & Ipilimu-mab *n* = 78	33.3%IIIA + 51.3%IIIB	43.2%	10.7	62.9%	NR^b^
		placebo *n* = 75	36%IIIA+48%IIIB	40.3%	14.5	66.4%	32.1
	ADRIATIC^9^	Durva-lumab *n* = 264	87.5%III	46.2%	16.6	68.0%	55.9
		Placebo *n* = 266	87.2% III	34.2%	9.2	58.5%	33.4
Immunotherapy followed by surgery or radiotherapy (our study)		ITT^a^ *n* = 40	95%III (40%IIIA + 40%IIIB + 15%IIIC)	42.5%	16.2	75.4%	NR^b^
		Surgery *n* = 21	95.2%III (57%IIIA + 28.6%IIIB + 9.5%IIIC)	61.9%	NR^b^	85.7%	NR^b^
		Radio-therapy *n* = 15	92.9%III (14.3%IIIA + 50%IIIB + 28.6%IIIC)	14.3%	13.59	42.9%	20.2

^a^ITT intention-to-treat population, ^b^NR not reached.

Biomarkers for the effectiveness of immunochemotherapy have remained elusive, with evidence supporting (and opposing) tumour mutation burden and PD-L1 expression^37,38^. By connecting baseline omics data and patient responsiveness, *PRSS8* was identified as a potential biomarker associated with poor response of immunochemotherapy in this study. PRSS8 is a glycosylphosphatidylinositol-anchored serine protease that is expressed in many human tissues^39,40^. PRSS8 plays important roles in epidermal barrier function^39,40^ and the regulation of glucose homeostasis^41^. *PRSS8* was reported to be associated with epithelial-mesenchymal transition in human bladder transitional cell carcinoma and non-small-cell lung cancer^42,43^. In this study, SCLC patients with high *PRSS8* expression were resistant to immunochemotherapy and had poor survival. These findings were also validated in several independent SCLC cohorts. Patients with higher expression of *PRSS8* mRNA showed enrichment of genes involved in estrogen response pathways, which might contribute to the worse therapeutic effect of immunochemotherapy^14,44^. Taken together, these findings indicate that the *PRSS8* might act as a prognostic biomarker in SCLC.

The TME directly influences the effectiveness of immunochemotherapy and, at the same time, is altered by immunochemotherapy^45^. By comparing the TME and immune repertoires between pretreatment and posttreatment samples, the dynamic molecular changes during induction therapy were investigated in this study. Transcriptional profiling has suggested that SCLC can be subdivided into subtypes of disease based on the differential expression of a small number of “master” TFs^46^. Thus, four distinct subtypes of SCLC defined by the predominant transcriptional regulatory mechanism operating in cancer cells have been described on the basis of the expression of *ASCL1*, *NEUROD1*, *YAP1*, and *POU2F3*^46^. Notably, the expression of these “master” TFs changed with induction therapy. *YAP1* expression emerges while *ASCL1* and *NEUROD1* expression decreases, resulting in a mixed tumour with some *YAP1* and *ASCL1* expressed side-by-side within the same tumour. In addition, the resulting ANPY categorization notably overlaps with, but is not identical to, the defining SCLC ANPI subtypes^10^. *YAP1* expression in SCLC defines a distinct subtype with a T-cell-inflamed phenotype, and the newly proposed SCLC-I subtype benefits the most from immunochemotherapy^10,47^. Our exploratory analysis revealed a significant increase in the percentage of SCLC-I subtype in posttreatment samples, revealing dramatic remodelling of the TME in SCLC patients after immunochemotherapy.

This study has several limitations. First, given the main purpose of exploring the feasibility of surgery, the increasing difficulty in surgery after radiotherapy, the low tolerant to CCRT, and the unknown AEs of CCRT combined with immunotherapy at the study design, we choose surgery after immunochemotherapy or sequential immuno-chemoradiotherapy. Moreover, our findings of this phase II trial with small samples size can only preliminarily explain the trend in the effectiveness of surgery after induced immunochemotherapy and act as a research reference for later studies. Furthermore, the effectiveness of sequential radiotherapy, concurrent radiotherapy, and surgery in the context of induced immunochemotherapy needs to be further compared in randomized, controlled phase III clinical trials. Additionally, patients were not randomly assigned to surgery or radiotherapy, and thus the outcomes of surgery and radiotherapy could not be compared. Subsequently, bioinformatic analysis was conducted without FDR correction and these findings need to be confirmed by larger independent studies.

In conclusion, surgery after induced anti-PD-L1 antibody (TQB2450) therapy and chemotherapy could be a safe and feasible treatment for patients with stage I-III SCLC. *PRSS8* was a potential biomarker for poor effectiveness of immunochemotherapy.

## Materials and methods

### Study design and participants

The LungMate-005 trial was an investigator-initiated, single-centre, phase II trial that was performed at Shanghai Pulmonary Hospital (Trial Registration: clinicaltrials.gov. Registration number: NCT04539977). The inclusion criteria were as follows: patients aged ≥ 18 years; tumour histologically or cytologically confirmed as SCLC; tumour radiologically confirmed at stage I‒III that can be safely treated with definitive radiation doses; patients with Eastern Cooperative Oncology Group (ECOG) performance status score of 0‒1; and patients with adequate organ function. The exclusion criteria were as follows: patients who received any systemic anti-tumour treatment for SCLC; patients with other malignancies; patients with active or previous autoimmune disorders, interstitial disease, and active serious infections; patients with multiple lung nodules that are too extensive or with tumour/nodal volume that is too large to be encompassed in a tolerable radiation plan.

All patients provided written informed consents before enrolment, and patients had the right to withdraw from the study at any time and for any reason. The protocol was approved by the clinical research ethics committee of Shanghai Pulmonary Hospital (L20-298-2).

Patients were scheduled to receive four cycles of 21-day-cycles induction therapy: TQB2450 (1200 mg, day 1), carboplatin (AUC5, day 1), and etoposide (100 mg/m^2^, days 1‒3). Patients underwent a systemic examination 3‒4 weeks after the fourth cycle of therapy. A multidisciplinary team (MDT) discussed the appropriate recommendations for surgery or radiotherapy. The criteria for surgical resection (as follows): (1) The surgical approach included segmentectomy, lobectomy, and sleeve resection but not pneumectomy. (2) If there was severe scarring of the hilar structure, and extensive resection was needed, radiotherapy should be chosen. (3) PET-CT indicated that lymph nodes (N2) were negative or that the diameter of the lymph nodes (N2) was smaller than 15 mm before surgery. (4) For patients who were staged as N3 positive at baseline, surgery was allowed only when lymph nodes (N3) were confirmed to be pathological negative by endobronchial ultrasound, supraclavicular lymph node puncture biopsy, and/or mediastinoscopy. (5) The patient wanted to receive surgery. Patients and their families then decide whether to receive surgery. Surgery or radiotherapy was performed 4‒6 weeks after the last dose of induction therapy. Radiotherapy was given at 54‒60 Gy in once-daily fractions of 1.8‒2 Gy for 30 days. The maintenance therapy (21-day-cycles) consisted of 2 cycles of TQB2450 (1200 mg, day 1), carboplatin (AUC5, day 1), and etoposide (100 mg/m^2^, days 1‒3) followed by 1 year of TQB2450. Treatment continued until disease progression, unacceptable toxicity, patient withdrawal, investigator decision to discontinue, or up to 1 year of treatment with TQB2450 (1200 mg, day 1). Dose reduction was permitted for carboplatin and etoposide, but not for TQB2450.

PET-CT and chest CT imaging were performed within 28 days before the first cycle of treatment to determine the TNM stage. Endobronchial ultrasound was performed to determine the pathological stage of the lymph node. Additional chest CT imaging was performed 3‒4 weeks after the completion of each 2 cycles. Radiologic response was assessed according to Response Evaluation Criteria in Solid Tumours version 1.1. Pathological examination was performed by two independent pathologists. PD-L1 expression was measured with a 22C3 pharmDx kit (Dako, Carpinteria, CA, USA). Primary tumours were assessed for the percentage of residual viable tumour (RVT) via routine haematoxylin and eosin staining after the operation. Tumours with no more than 10% RVT were considered to have a MPR, and tumours together with lymph nodes without RVT were considered to have a PCR.

### Outcomes

The primary endpoint was the ORR. Secondary endpoints included the incidence of AEs, EFS, PFS, OS, MPR, and PCR. The exploratory outcomes included biomarkers that could predict the effectiveness of immunochemotherapy and molecular changes in the TME.

The ORR was defined as the proportion of patients with a confirmed complete or partial response. The duration of overall response was defined as the first complete or partial response to the first disease progression or death. DFS was defined as the time from surgery to disease progression or death, whichever happened first (only for those who received surgery). PFS was defined as the time from diagnosis to disease progression or death, whichever happened first (only for those who did not receive surgery). EFS was defined as the time from diagnosis to recurrence (for patients who received surgery), disease progression (for patients who did not receive surgery), or death, whichever happened first. Safety was monitored for AEs from the time of informed consent to 90 days after the last administration of the study medication according to the National Cancer Institute Common Terminology Criteria for Adverse Events version 5.0.

### DNA sequencing and data processing

In this study, 12 baseline (pretreatment) samples and 5 posttreatment samples were collected and successfully subjected to DNA sequencing. Genomic DNA was extracted from tumours using a QIAamp Fast DNA Tissue Kit (QIAGEN) according to the manufacturer’s protocol. Total DNA was quantified by a Qubit 2.0 fluorometer (Life Technologies) and NanoDrop 2000 (Thermo Fisher Scientific), and the integrity of the DNA was assessed by TapeStation (Agilent Technologies). For Illumina library construction, the genomic DNA was fragmented to an average size of 180‒280 bp using a Covaris focused ultrasonicator. Then, WES libraries were prepared and captured using the Agilent SureSelect Human All Exon V6 kit (Agilent Technologies) following the manufacturer’s instructions. The DNA library with 150 bp paired-end reads was sequenced on the Illumina NovaSeq 6000 platform.

To detect single-nucleotide variants (SNVs) and small insertions/deletions (INDELs), the Genome Analysis Toolkit (GATK, v4.0.6.0) best practice guidelines were used. After excluding low-quality reads, qualified paired-end WES sequencing reads were aligned to the human reference genome (hg38) with BWA MEM (v0.7.15)^48^. The resulting BAM files were further processed with Picard tools to remove PCR duplicates. SNVs and INDELs were detected using MuTect2 in tumour-only mode with a panel of normal samples created from adjacent normal samples from another study, which was deposited in HRA003419. Then we filtered out short tandem repeat regions downloaded from the UCSC table browser and annotated them using Funcotator from GATK. Oncoplot plots were generated using the “Maftools” R package^49^. TMB was calculated via “tmb” function in “maftools” R package with capture size of 60.

### RNA sequencing and data processing

In this study, 17 baseline samples and 21 posttreatment samples were collected and successfully subjected to RNA sequencing. Total RNA from fresh-frozen tissues was extracted with TRIzol. Sequencing libraries were generated using a NEBNext Ultra RNA Library Prep Kit for Illumina, and index codes were added to attribute sequences to each sample. The libraries were pooled and paired-end sequencing (2 × 150 bp reads) was performed using an Illumina NovaSeq 6000. After RNA-seq, the raw fastq files were trimmed via fastp (v0.20.1)^50^ and aligned to the GRCh38 reference genome by STAR (v2.7.6a)^51^ with default settings. After obtaining the BAM files, the read counts were summarized by featureCounts (v2.0.1)^52^. RNA sequencing (RNAseq) data were calculated as counts per million (CPM) and log-transformed and TMM-normalized. Batch effects were adjusted using the “combat” function in the sva package. The SCLC “ANPI” subtype was defined as previously described^10^. SCLC-I subtype is characterized by low expression of ASCL1, NEUROD1, and POU2F3. The SCLC “ANPY” subtype was identified based on the differential expression of genes encoding the TFs: ASCL1, NEUROD1, POU2F3, and YAP1, and the single gene with the highest expression was used to define the SCLC-A, SCLC-N, SCLC-P and SCLC-Y subtypes^46^.

### Differentially expressed gene analysis

We used DESeq2^53^ to calculate differential gene expression between sample groups. The DESeq2 genes were profiled according to model gene count expression data, and the Log_2_(fold change) was calculated to estimate the effect size and to represent gene changes between comparison groups. Two-sided Wald test statistics were computed to examine differential expression across the comparison groups. Genes with |Log_2_(fold change)| > 1 and Wald test *P* < 0.05 were defined as differentially expressed genes. We drew volcano plots to visualize the differential gene expression results.

### Gene set enrichment analysis (GSEA)

For GSEA, the results for all protein-coding genes were ranked by Log_2_(fold change) and evaluated with the ‘GSEA’ algorithm^54^. ‘Hallmark’ and ‘KEGG’ gene sets were acquired from MSigDb. We filtered GSEA results based on the criterion of *P* < 0.05 and visualized the pathways as candidates based on the normalized enrichment score.

### Tumour microenvironment estimation

For immune repertoire analysis, the TRUST4 (v1.0.0)^55^ algorithm was applied to evaluate the immune repertoire and to extract T-cell receptor (TCR) and B-cell receptor (BCR) complementarity-determining region 3 (CDR3) sequences. The infiltration of immune cells was evaluated by “MCPcounter (v1.2.0)”.

### Biomarker exploration

In the analysis of the predictive markers in this study, we employed a rigorous screening process: 1, The genes were upregulated in poor-responder (log_2_FC < ‒1 and *P* < 0.05) according to both the DEseq2 and Wilcoxon results (*n* = 117); 2, The genes were a negative prognostic indicator in our cohort and the Roper’s immunotherapy cohort (HR > 1 and log rank *P* < 0.1) (*n* = 1); Finally, we focused on PRSS8 as the sole gene of interest. Furthermore, we employed an independent immunotherapy IMpower133 cohort to validate the predictive value of PRSS8 to immunotherapy effectiveness. The process to determine low or high PRSS8 expression groups (the distribution of expression and thresholds) were shown in Supplementary Fig. S7.

### Immunohistochemistry analysis

The sections were subjected to IHC staining according to standard procedures with anti-PRSS8 antibody (Abcam, ab203879), anti-NEUROD1 antibody (Abcam, 109224), anti-ASCL1 antibody (BD, BD556604), anti-YAP1 antibody (CST, 17074S), anti-DLL3 antibody (TFS, PA5-23448), and anti-HES1 antibody (CST, 11988).

### Statistical analysis

The ORR, survival outcomes, and incidence of AEs were analysed for all enrolled patients (*n* = 40). Pathological response and surgical outcomes were only evaluated in patients who underwent surgery (*n* = 21).

We defined a treatment as unfeasible if its ORR was less than 70% (the ORR of concurrent chemoradiotherapy^1,7,8,26^). We used the Kaplan‒Meier method to estimate DFS, EFS, and OS. The Wilcoxon rank sum test was further applied to compare the differences in expression between sample groups. To separate patients into low- and high-PRSS8-expression groups, a cut-off was generated based on the association between gene expression and survival data using the “surv_cutpoint” function in the survminer package. AUC was generated using “roc” function in “pROC” R package. ACC was generated using “reportROC” R package.

The *P* values were two-sided, and the significance level was set at 0.05 for all analyses. R (v 4.3.1) and SPSS (IBM, v 26) were used for all the statistical analyses.

## Supplementary information

supplementary information

## Data Availability

Source data are provided with this paper. All RNA-seq and WES data of nine samples generated during the current study were deposited in the Genome Sequence Archive database under accession number HRA005288. Other WES data from eight samples were deposited in the Genome Sequence Archive database under accession number HRA003419. The code used in this study has been deposited at https://github.com/zero123321/Surgery-after-Induced-PD-L1-Inhibitor-TQB2450-Treatment-and-Chemotherapy-for-Limited-stage-SCLC.
