# Supplementary material for: Surgery after induced anti-PD-L1 therapy and chemotherapy for stage I‒III small-cell lung cancer: a phase 2 trial (LungMate-005)
Source: Cell Discov. 2025 Nov 25;11:95. doi: 10.1038/s41421-025-00838-5 (PMC12647595; doi:10.1038/s41421-025-00838-5)
Supplement: Supplementary file 1 — supplementary information [file 41421_2025_838_MOESM1_ESM.pdf]

Supplementary Figures

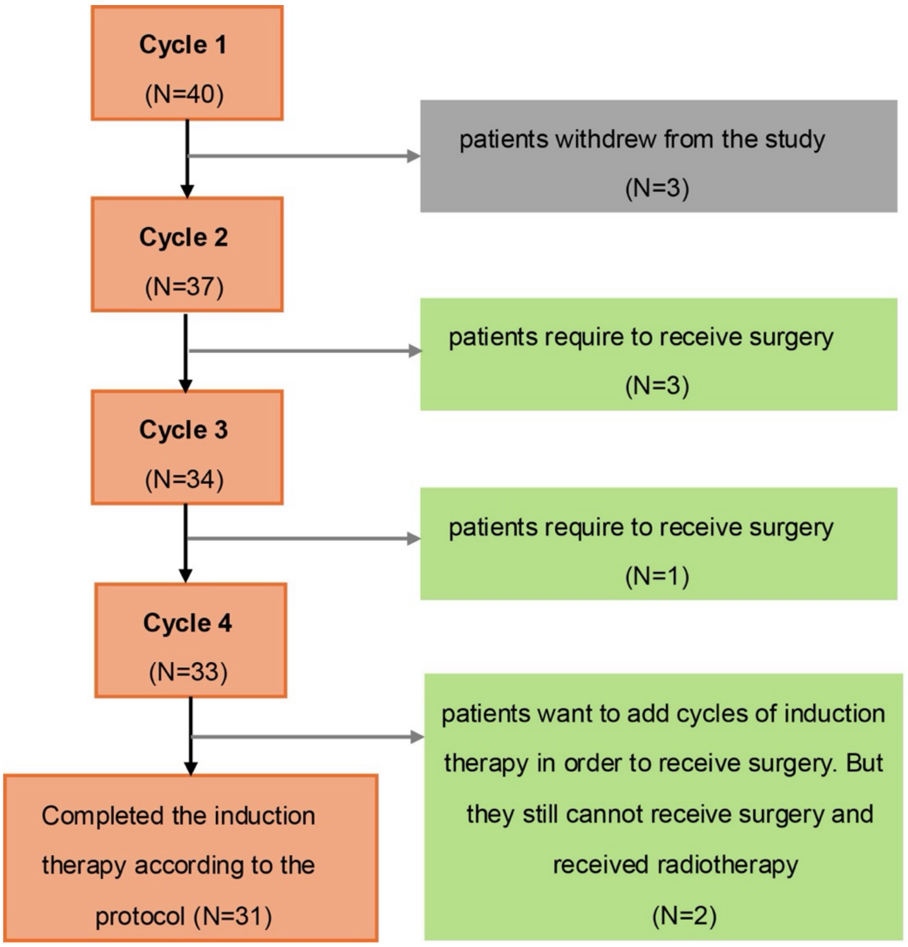

Supplementary Fig. S1 The adherence to protocol-specified regimen.

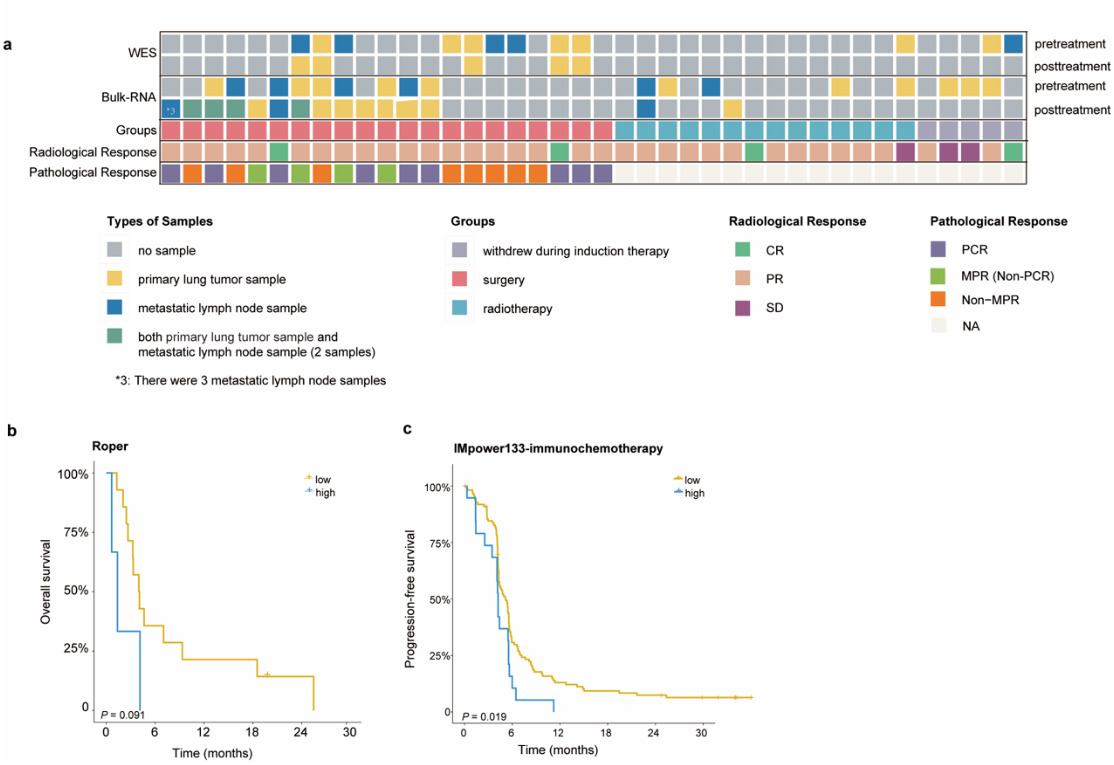

Supplementary Fig. S2 PRSS8 and induction therapy.

a, Map of RNA and WES sample distribution. b, The difference in OS between patients with high and low *PRSS8* mRNA expression in Roper's cohort (N=17). c, The difference in EFS between patients who received immunochemotherapy with high and low *PRSS8* mRNA expression in IMpower133 cohort (N=132).

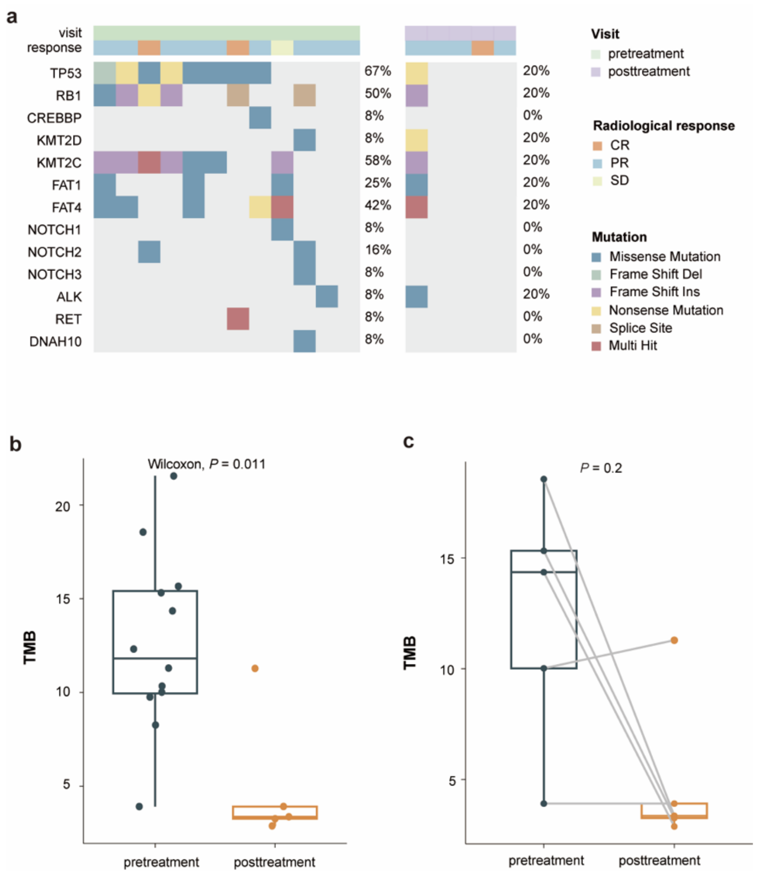

Supplementary Fig. S3 Genomic characteristics and induction therapy.

a, Mutational profiles in pretreatment (N=12) and posttreatment (N=5) samples. b-c, Changes in the tumour mutation burden with induction therapy in all samples (b) and patients with matched longitudinal samples (c).

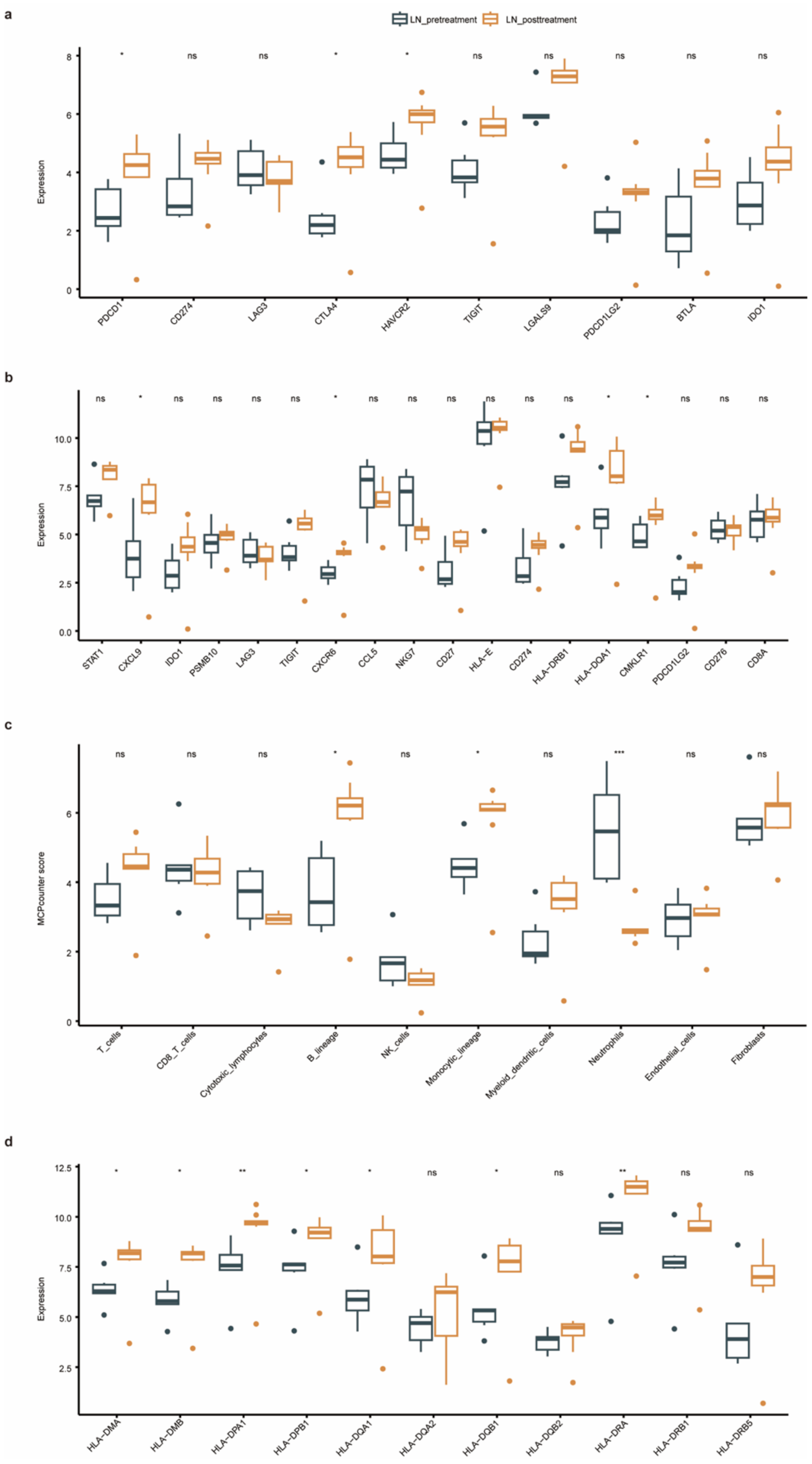

Supplementary Fig. S4 Changes in the tumour microenvironment between pretreatment (N=6) and posttreatment (N=9) LN samples.

a, Increased immune checkpoints expression in posttreatment LN samples. b, Increased T-cell effector genes expression in posttreatment LN samples. c, Immune cells infiltration changes along with treatment d, Increasing MHC-II members expression in posttreatment LN samples.

* The units for the quantified expression are log2(CPM+1).

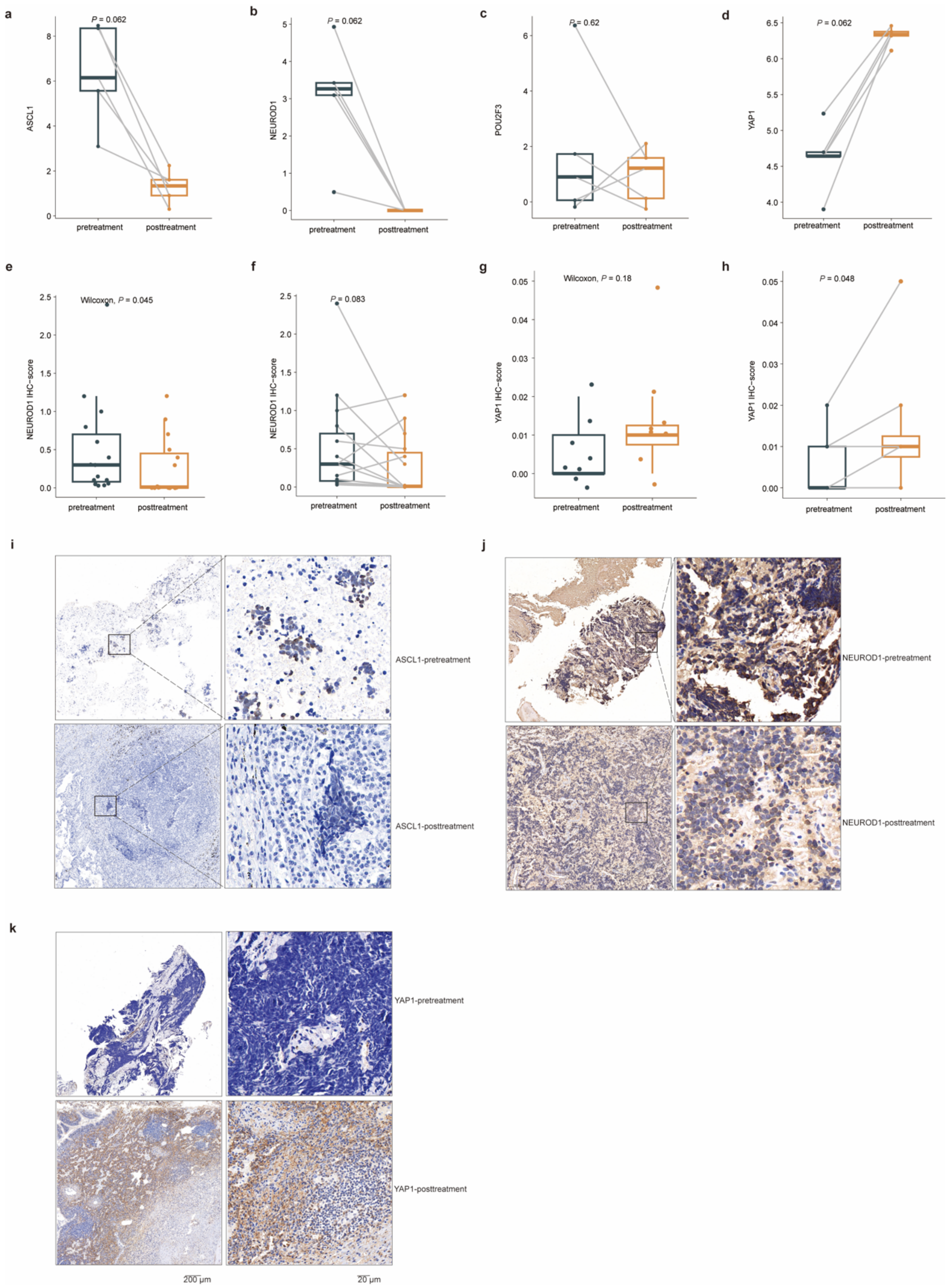

Supplementary Fig. S5 ANPY expression changes along with treatment.

a-d, Key TFs changes along with treatment. *ASCL1* (a), *NEUROD1* (b), *POU2F3* (c) and *YAP1* (d) RNA expression changes along with treatment in patients with matched longitudinal samples. e-f, IHC expression

changes of NEUROD1 along with treatment via unpaired (e) and paired (f) Wilcoxon test. g-h, IHC expression changes of YAP1 along with treatment via unpaired (g) and paired (h) Wilcoxon test. i, Representative images for down-regulation of ASCL1 after treatment. j, Representative images for down-regulation of NEUROD1 after treatment. k, Representative images for up-regulation of YAP1 after treatment.

* The units for the quantified *ASCL1*, *NEUROD1*, *POU2F3* and *YAP1* expression in Figure S4a-d are log2(CPM+1).

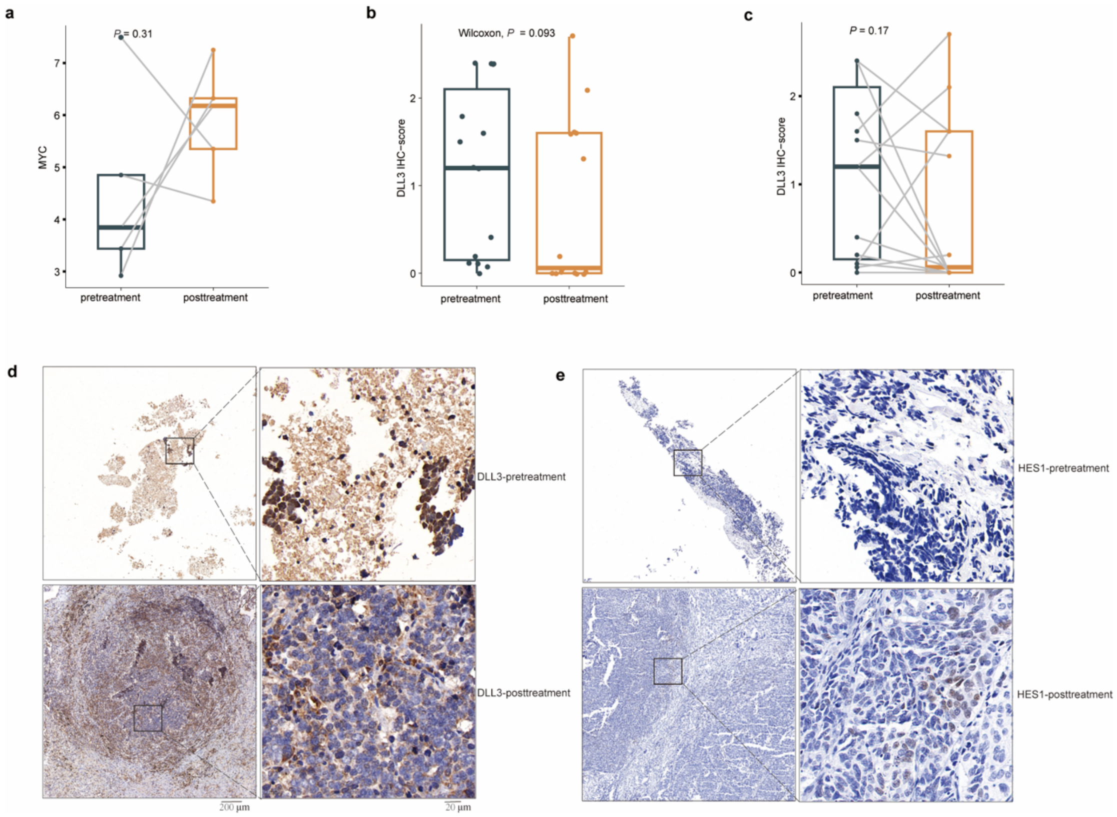

Supplementary Fig. S6 Potential mechanisms for neuroendocrine phenotype transition.

a, High *MYC* RNA-seq expression after treatment in patients with matched longitudinal samples. b-c, DLL3 IHC expression changes along with treatment via unpaired and paired Wilcoxon test. d, Representative images for down-regulation of DLL3 after treatment. e, Representative images for up-regulation of HES1 after treatment.

* The unit for the quantified *MYC* expression in Figure S5a is log2(CPM+1).

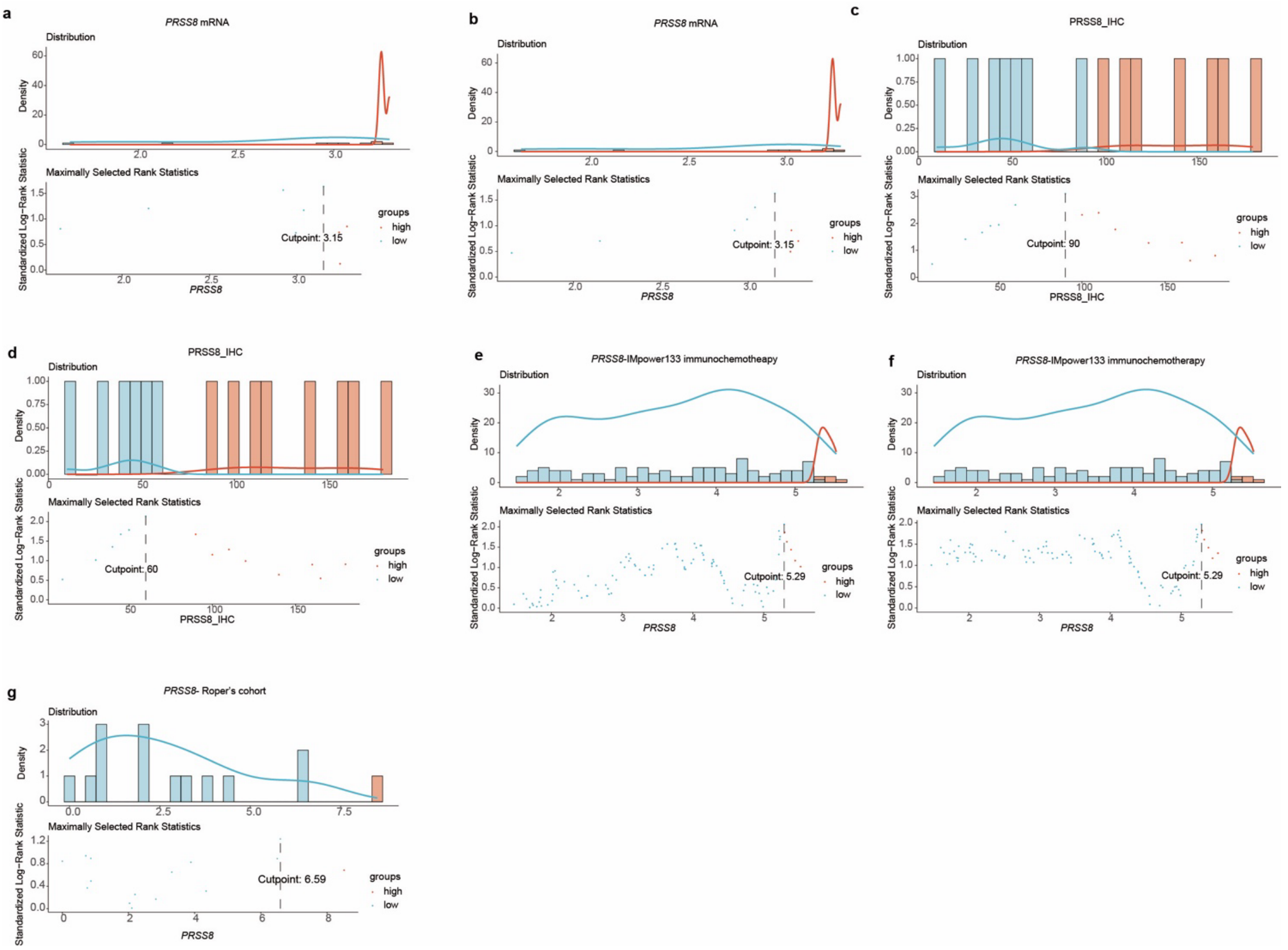

Supplementary Fig. S7 The distribution of expression and thresholds of PRSS8 in the discovery and validation cohorts.

a-b, The distribution of expression and thresholds of PRSS8 mRNA expression in analysing OS(a) and EFS(b) in our cohort. c-d, The distribution of PRSS8 IHC expression and thresholds in analysing OS(c) and EFS(d) in our cohort. e-f, The distribution of PRSS8 mRNA expression and thresholds of in analysing OS(e) and PFS(f) for patients receiving immunotherapy and chemotherapy in Impower133 cohort. g, The distribution of PRSS8 mRNA expression and thresholds of in analysing OS in Roper's cohort.

Program title: Surgery or Radiotherapy After PD-L1 Inhibitor (TQB-2450) and Chemotherapy Induction Therapy in Patients with Limited-stage Small-cell Lung Cancer

Program Number: SCLC-TQB2450-001(LungMate-005)

Version number: 1.1

Release date: December 27, 2020

Experimental drug: TQB2450 injection

Drug supplier: Chia Tai Tianqing Pharmaceutical Group Co.,Ltd. Shanghai. China.

Sponsor: Department of Thoracic Surgery, Shanghai Pulmonary Hospital

Confidentiality statement

The information contained in this document, in particular unpublished data, is the property of the sponsor of this study and is provided to you as a researcher, potential researcher or consultant in confidence for you and your research team and for the independent Ethics Committee/Institutional Review Board. It must be made clear that such information is not to be disclosed to others without the written authorization of the sponsor, except in the case of obtaining informed consent from patients who may be taking the medication.

Sign of principal investigator: ________________________

Date: ________________________

Researcher information

Principal Investigator: Peng Zhang, Deputy Chief Physician, Professor

Tel: 021-65115006-1008

Email: zhangpeng1121@tongji.edu.cn

507 Zhengmin Road, Yangpu District, Shanghai 200433, China

Contents

1.Background 5
1.1 Small cell lung cancer 5
1.2 Current treatment for SCLC 5
1.3 Treatment of extensive stage small cell lung cancer 6
1.4 Immunotherapy: PD-L1 8
1.5 PD-L1 inhibitor: TQB2450 8
1.6 PD-L1 inhibitor in treating SCLC 8
1.7 Research background and aim 9
2. Research Objectives and Endpoints 10
2.1 Research Objectives 10
2.1.1 Primary Objectives 10
2.1.2 Secondary Objectives 10
2.1.3 Exploratory Objectives 10
2.2 Research Endpoints 10
2.2.1 Primary Endpoints 10
2.2.2 Secondary Endpoints 10
2.2.3 Exploratory Endpoints 11
3. Study Design 11
3.1 Overview of Study Design 11
3.1.1 Induction Therapy Period (4 cycles, 3 weeks per cycle): 11
3.1.2 Surgery or Radiotherapy: 11
3.1.3 Maintenance Therapy (every 3 weeks): 12
3.2 Sample size 12
3.3 Enrolment Criteria 12
3.3.1 Inclusion Criteria 12
3.3.2 Exclusion Criteria 13
3.4 Estimated research duration 14
4. Treatment 14

4.1 Treatment Protocol 14
4.1.1 Induction Therapy 14
4.1.2 Surgery or Radiotherapy 14
4.1.3 Maintenance Therapy 14
4.2 Preparation and Storage 15
4.2.1 TQB2450 15
4.2.2 Chemotherapy Drugs 16
4.3 Dosage, Administration, and Compliance 16
4.4 Drug Dose Adjustment 17
4.4.1 TQB2450 Dose Adjustment 17
4.4.2 Chemotherapy Dose Adjustment 18
5. Combination Therapy 20
5.1 Permitted Combination Drugs/Treatments 20
5.2 Prohibited or Restricted Combination Drugs/Treatments 21
6. Research Assessment and Procedures 21
6.1 Screening 22
6.2 Confirmation of Eligibility for Enrollment 22
6.3 Assessment of safety 23
6.5 Follow-up Period 25
6.6 Visit Windows 25
7. Statistical methods and sample size 26
7.1 Statistical Analysis 26
7.2 Safety Analysis 26
7.3 Effectiveness Analysis 26
8. Reference 28
Appendix 1. Evaluation flow sheet 31

1.Background

1.1 Small cell lung cancer

Primary bronchogenic carcinoma is one of the most prevalent malignant tumors, with lung cancer accounting for the highest incidence (12.9%) and mortality (23.5%) among all malignancies, according to the updated statistics in 2019 [1]. In China, lung cancer had the highest incidence (19.6%) and mortality (26.0%) rates among all malignancies, exhibiting a notable increase compared to the rates in 2007 (13.0% and 18.0%, respectively) [2-4]. Small cell lung cancer (SCLC) constitutes an aggressive subtype, originating from neuroendocrine tumors derived from the bronchial mucosal epithelium, comprising approximately 15%-20% of lung cancer cases [5, 6]. Currently, SCLC is classified according two staging systems: the 8th edition TNM staging or the commonly used VA staging system. According to the commonly used VA staging system, limited-stage small cell lung cancer (LS-SCLC) involved tumors confined to the same side of the thoracic cavity within a manageable radiation field (30%-40% of SCLC cases), and extensive-stage small cell lung cancer (ED-SCLC) encompassed tumors extending beyond the same side of the thoracic cavity, with distant metastases, malignant pleural effusion, or pericardial effusion [7]. Smoking is responsible for 98% of SCLC cases, characterized by high malignancy, strong invasiveness, rapid disease progression, and early occurrence of distant metastases [8, 9]. Despite SCLC was sensitive to chemotherapy and radiotherapy, SCLC has short remission period and high recurrence rates. Most SCLC patients are elderly, with a history of smoking or heavy smoking, often accompanied by various chronic comorbidities. Consequently, treatment outcomes are often compromised, leading to an overall poor prognosis, with a median overall survival (mOS) not exceeding one year and a two-year survival rate below 5%.

1.2 Current treatment for SCLC

Since 1980, multiple clinical trials have consistently demonstrated that platinum-based combinations, particularly platinum combined with etoposide in treating small cell lung cancer (SCLC) has higher complete response rates and improved prognosis [10, 11]. These findings have solidified the foundational role of platinum-based regimens in the therapeutic approach for SCLC. A phase III clinical trial published in 2002 further substantiated the favorable outcomes associated with platinum-based therapy, specifically cisplatin plus etoposide (EP), in patients with limited-

stage SCLC (LS-SCLC) [12]. SCLC exhibits high sensitivity to radiotherapy, with guidelines currently recommending chemotherapy (EP/EC) in combination with thoracic radiotherapy as the preferred treatment for LS-SCLC [6]. A meta-analysis demonstrated that concurrent chemoradiotherapy significantly reduces the local recurrence rate by 25%-30% and increases the 2-year survival rate by 5%-7% compared to chemotherapy alone, underscoring the crucial role of radiotherapy in the treatment of LS-SCLC [13]. The role of surgery in SCLC remains controversial, with early randomized studies showing no benefit from surgical intervention [14, 15]. However, these studies conducted in the early years. Results may be different affected by the current novel drugs and surgical techniques. Subsequent retrospective studies since 2000 have suggested potential benefits of surgery for early or limited-stage SCLC patients [16-19]. Additionally, multiple studies have indicated that chemotherapy followed by surgery significantly improves the prognosis of early or limited-stage SCLC patients, suggesting potential benefits for LS-SCLC [20-22]. However, the debate persists regarding whether surgery or neoadjuvant chemotherapy followed by surgery is the preferred approach for LS-SCLC patients. Research by Fujimori et al. found that stage I-II and stage III SCLC patients undergoing neoadjuvant chemotherapy and surgery had 3-year survival rates of 73.3% and 42.9%, respectively [23]. A prospective study by Veronesi et al. at a single center revealed that SCLC patients achieving complete remission and those staged as pathological stage I after neoadjuvant chemotherapy could benefit from surgery [24].

Clinical trials in treating SCLC					
Trials	Stage	Treatment	Patient Number	5 year-OS	P value
Schreiber[16]	LS-SCLC	Surgery vs non-surgery	863 vs 13316	42% vs 13%	<0.001
Luchtenborg[17]	LS-SCLC	Surgery vs non-surgery	465 vs 45383	31% vs 3%	<0.001
James[18]	I	Surgery +RT vs surgery	38 vs 205	57.1% vs 50.3%	0.71
Weksler[19]	I - II	Surgery vs non-surgery	893 vs 2666	26.9% vs 7%	<0.001
Yang[20]	T1-2N0M0	surgery＋chemotherapy (radiotherapy) vs surgery	544 vs 388	52.7% vs 40.4%	<0.01
Tsuchiya[21]	I － IIIA	Surgery +chemotherapy	35 vs 26	70% vs 38.5%	0.10
Yang[22]	T1-2N0M0	surgery+ chemotherapy vs chemoradiotherapy	501 vs 501	47.6% vs 29.8%	<0.01

1.3 Treatment of extensive stage small cell lung cancer

Currently, both the National Comprehensive Cancer Network (NCCN) and the Chinese Society

of Clinical Oncology (CSCO) recommend platinum-based chemotherapy (cisplatin or carboplatin) in combination with the topoisomerase II inhibitor (etoposide) as the standard first-line treatment for extensive-stage small cell lung cancer (ES-SCLC), administered for four cycles. In Japan, irinotecan plus platinum-based chemotherapy was commonly used [6, 25]. In the mid-1980s, clinical studies on the first-line treatment for ES-SCLC with a combination of cisplatin and etoposide demonstrated a complete response (CR) rate exceeding 40%, with a median survival time of approximately 9 months [26]. Subsequent randomized studies with small number of eligible patients indicated similar effectiveness of treatment regimens containing either carboplatin or cisplatin in SCLC patients [27, 28]. A meta-analysis comparing cisplatin and carboplatin treatment regimens for SCLC patients from four randomized studies revealed no significant differences in response rates (67% vs. 66%), progression-free survival (PFS) (5.5 months vs. 5.3 months), or overall survival (OS) (9.6 months vs. 9.4 months) between patients treated with cisplatin and those treated with carboplatin, suggesting equivalent effectiveness of these two drugs in treating SCLC. Multiple studies on the combination of cisplatin or carboplatin with etoposide (at various doses) consistently yielded concordant results, as shown in the table below.

Clinical trials of using platinum + etoposide in treating ES-SCLC				
Trials	platinum	ORR	中位PFS (月)	中位POS (月)
Roth [30]	cisplatin	61%	4.3	8.6
Pujol [31]	cisplatin	61%	6.3	9.3
Noda [32]	cisplatin	68%	4.8	9.4
Eckardt [33]	cisplatin	69%	6.3	10.1
Hanna [34]	cisplatin	44%	4.6	10.2
Okamoto [28]	cisplatin	73%	4.7	9.9
Okamoto [28]	carboplatin	73%	5.2	10.6
Rudin [35]	carboplatin	52%	5.4	10.6
Socinski [36]	carboplatin	60%	7.6	10.6
Nagel [37]	carboplatin	67%	7.0	11.0
Schmittel [38]	carboplatin	52%	6.0	9.0

Despite the impressive response rates was observed in first-line chemotherapy, a majority of patients with extensive-stage small cell lung cancer (ES-SCLC) inevitably develop chemotherapy resistance, leading to a poor prognosis. Progression-free survival (PFS) generally remains less than 6 months, and the median overall survival is still less than 1 year. Therefore, it is imperative to explore new therapeutic options for ES-SCLC patients. Immunotherapeutic agents, including

antibodies that modulate the activity of immune cells, offer a potential alternative treatment approach with the potential to improve the prognosis of such patients.

1.4 Immunotherapy: PD-L1

Programmed Death-Ligand 1 (PD-L1), also known as CD274 or B7-H1, is a 40 kD transmembrane protein which is encoded by the CD274 gene and is induced on the surface of various immune cells [39]. The expression of PD-L1 in SCLC was reported in several research [40], The interaction between PD-1 and PD-L1 may contribute to immune evasion in SCLC, thereby promoting tumor development. This hypothesis is supported by independent studies. PD-L1 expressed on tumor cells increases the apoptosis of activated tumor-specific T cells in vitro, while PD-L1 expression protects tumor cells from apoptosis induced by effector T cells [41]. Preliminary clinical data suggest that PD-1 has a blocking effect on both PD-L1-positive and PD-L1-negative tumors, indicating its potential role at the level of immune initiation and the tumor microenvironment [42]. This suggests that the expression of PD-L1 on antigen-presenting cells may limit the anti-tumor response. Therefore, both PD-L1-positive and PD-L1-negative tumors may benefit from immunotherapy.

1.5 PD-L1 inhibitor: TQB2450

TQB2450 is a monoclonal antibody that binds to PD-L1, developed by the Chia Tai Tianqing Pharmaceutical Group Co.,Ltd. It can block the interaction between PD-L1 and PD-1 and B7.1 receptors, releasing the inhibition of the PD-L1/PD-1-mediated immune response, including the activation of antibody-independent cytotoxic effects in the anti-tumor immune response. This novel PD-L1 inhibitor fully humanized monoclonal antibody belongs to the innovative monoclonal antibodies and is classified as a Class I therapeutic biological product. In October 2017, it obtained approval from the China Food and Drug Administration for clinical research (CFDA: 2017L04914). TQB2450 has completed phase I clinical trials which assessed tolerability and pharmacokinetic in treating advanced malignant tumors.

1.6 PD-L1 inhibitor in treating SCLC

In recent years, immunotherapy has become a major focus of researchers in the field of cancer. Researchers hold promise focus on it due to its distinct mechanism of action. Given SCLC has heterogeneity, genomic instability, and high tumor mutation burden, it may be sensitive to

immunotherapy. Thus, numerous clinical trials was performed to explore the effectiveness and safety of immunotherapy in treating SCLC. Current studies mainly focus on extensive-stage SCLC (ES-SCLC) patients, predominantly employing combination therapies, including immunotherapy with chemotherapy, immunotherapy with targeted drugs, and combinations of immunotherapy with other modalities. Several research outcomes indicate that PD-L1 inhibitor and other therapy combination therapy may outperform the standard treatment for SCLC, with PD-L1 inhibitor combined with platinum and etoposide chemotherapy improving the prognosis of ES-SCLC patients [43, 44]. Results from a multicenter, randomized, double-blind phase III study (CASPIAN) showed that the PD-L1 inhibitor Durvalumab, combined with platinum and etoposide, significantly improved the median overall survival (OS) of ES-SCLC (13.0 months vs. 10.3 months) [43]. Another multicenter, randomized, double-blind phase III study (IMpower133) demonstrated that the PD-L1 inhibitor Atezolizumab, combined with carboplatin and etoposide, significantly extended the median OS (12.3 months vs. 10.3 months) and median PFS (5.2 months vs. 4.3 months) in ES-SCLC [44]. Based on these data, the latest NCCN guidelines recommend Atezolizumab in combination with carboplatin and etoposide as the first-line treatment for ES-SCLC, supporting the incorporation of immune checkpoint inhibitors into the general concept of standard chemotherapy [6]. There is currently no research exploring the effectiveness of PD-L1 inhibitor in treating limited-stage SCLC (LS-SCLC).

1.7 Research background and aim

Currently, the standard first-line treatment for ES-SCLC is platinum (cisplatin or carboplatin) combined with etoposide. Despite a certain response rate in first-line chemotherapy, the prognosis for ES-SCLC patients remains poor, with a median OS of approximately 9-11 months. PD-L1 inhibitor as a first-line treatment for SCLC patients have demonstrated controllable safety profiles and promising efficacy, including increased response rates, prolonged median response duration, and extended OS. As a novel and innovative anti-PD-L1 fully humanized monoclonal antibody, TQB2450 may exhibit effectiveness in the clinical application of small cell lung cancer. In preliminary phase I clinical trials, TQB2450 has effect on treating SCLC and requires further evaluation of its effectiveness and safety. Simultaneously, investigations into the effects of TQB2450 in treating LS-SCLC need to be further explored.

2. Research Objectives and Endpoints

2.1 Research Objectives

2.1.1 Primary Objectives

Evaluate the objective response rate (ORR) according to RECIST v1.1 in the intention-to-treat (ITT) population, as a measure of effectiveness of TQB2450 + platinum + etoposide.

2.1.2 Secondary Objectives

Assess the effectiveness of TQB2450 + platinum + etoposide according to RECIST v1.1 in the ITT population, index including progression-free survival (PFS), disease-free survival (DFS), event-free survival (EFS), and overall survival (OS)

Evaluate the major pathological response (MPR) and pathological complete response (PCR) after induced TQB2450 + platinum + etoposide in operable limited-stage SCLC based on the assessment recommended by the International Association for the Study of Lung Cancer (IASLC).

Measure disease control rate (DCR) according to RECIST v1.1 in the ITT population.

Assess the incidence of serious adverse events (SAE) during treatment according to the National Cancer Institute Common Terminology Criteria for Adverse Events (NCI-CTCAE) v5.0.

Evaluate the impact of TQB2450 on health-related quality of life (HRQoL) using psychological assessment and quality of life questionnaires.

2.1.3 Exploratory Objectives

Explore potential predictive biomarkers in archived and/or fresh tumor tissue, including but not limited to programmed cell death-ligand 1 (PD-L1) expression determined by immunohistochemistry (IHC), gene expression profile (GEP), tumor mutation burden (TMB), and changes in the tumor microenvironment. To assess their correlation with treatment response or resistance mechanisms.

2.2 Research Endpoints

2.2.1 Primary Endpoints

Objective Response Rate (ORR): Defined as the proportion of patients achieving partial response (PR) or complete response (CR) according to RECIST v1.1 in ITT population.

2.2.2 Secondary Endpoints

Safety: Incidence of serious adverse events (SAE) during treatment according to NCI-CTCAE

v5.0.

Event-Free Survival (EFS): Time from patient diagnosis to the first occurrence of disease progression, postoperative relapse, or death for any reason, whichever occurs first.

Progression-Free Survival (PFS): Time from patient diagnosis to the first occurrence of disease progression or death for any reason, whichever occurs first.

Disease-Free Survival (DFS): Time from surgery to the first occurrence of postoperative relapse or death for any reason, whichever occurs first.

Overall Survival (OS): Time from patient diagnosis to death for any reason.

Pathological Complete Response (PCR) Rate: Proportion of patients with no visible residual tumor cells in tumor and lymph nodes based on pathological assessment.

Major Pathological Response (MPR) Rate: Proportion of patients with residual tumor cells visible on HE staining occupying ≤10% of the resected tumor.

Disease Control Rate (DCR): Proportion of patients with best overall response of CR, PR, or stable disease (SD) according to RECIST v1.1.

HRQoL: Impact of TQB2450 on health-related quality of life assessed using psychological evaluation and quality of life questionnaires.

2.2.3 Exploratory Endpoints

Biomarkers: Explore biomarkers in archived and/or fresh tumor tissue related to prognosis. such as PD-L1 expression, GEP, TMB, and changes in the tumor microenvironment. Assess their association with treatment response or resistance mechanisms.

3. Study Design

3.1 Overview of Study Design

This study comprises screening, treatment, safety follow-up, and survival follow-up periods, with the treatment period consisting of the induction therapy period and maintenance therapy period.

3.1.1 Induction Therapy Period (4 cycles, 3 weeks per cycle):

TQB2450 + platinum + etoposide

3.1.2 Surgery or Radiotherapy:

Patients who meet specific conditions and express a strong desire for surgery may undergo surgery. Otherwise, they will receive radiotherapy.

Criteria for Acceptable Surgical Treatment:

(1) Surgical approaches included segmentectomy, lobectomy, and sleeve resection, rather than pneumectomy.

(2) If severe scarring of the hilar structure appeared and extensive resection was required, radiotherapy should be chosen.

(3) PET-CT indicates that N2 lymph nodes were negative, or the diameter of N2 lymph nodes was smaller than 15mm before surgery.

(4) For patients who staged at N3 at baseline, surgery would be allowed only when N3 lymph nodes were confirmed as negative by endobronchial ultrasound, supraclavicular lymph node puncture biopsy, or/and mediastinoscopy.

(5) Patient desires to receive surgery.

Radiotherapy was given as 54-60 Gy in once-daily fractions of 1.8-2 Gy for 30 days.

3.1.3 Maintenance Therapy (every 3 weeks):

All enrolled patients may receive an additional 2 cycles of TQB2450 + platinum + etoposide, followed by TQB2450 for up to 1 year.

The research flow is as follows:

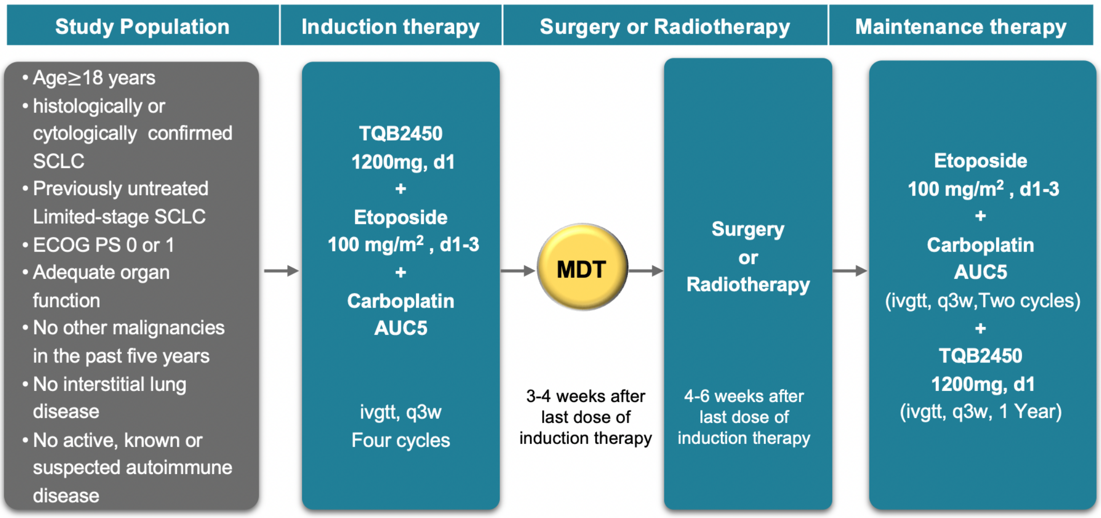

3.2 Sample size

About 40 participants will participate in this exploratory phase II trial.

3.3 Enrolment Criteria

3.3.1 Inclusion Criteria

A. The patient shall sign the Informed Consent Form.
B. Aged 18 ≥ years.
C. Histological or cytological diagnosis of SCLC by needle biopsy, and extensive stage or limited stage (local advanced) confirmed by imagological examinations.
D. Eastern Cooperative Oncology Group (ECOG) performance-status score of 0 or 1.
E. Life expectancy is at least 12 weeks.
F. At least 1 measurable lesion according to RECIST 1.1.
G. Patients with good function of other main organs (liver, kidney, blood system, etc.):
    - ANC count ≥1.5×10^9/L, platelet count ≥100×10^9/L, hemoglobin ≥90 g/L;
    - the international standard ratio of prothrombin time (INR) and prothrombin time (PT) < 1.5 times of upper limit of normal (ULN);
    - partial thromboplastin time (APTT) ≤1.5×ULN;
    - total bilirubin ≤1.5×ULN;
    - alanine aminotransferase (ALT) aspartate aminotransferase (AST) ≤2.5×ULN, or ALT and AST ≤5×ULN in the patients with liver metastatic tumor.
H. Fertile female patients must voluntarily use effective contraceptives not less than 120 days after chemotherapy or the last dose of TQB2450 (whichever is later) during the study period, and urine or serum pregnancy test results within 7 days prior to enrollment are negative.
I. Unsterilized male patients must voluntarily use effective contraception during the study period not less than 120 days after chemotherapy or the last dose of TQB2450 (whichever is later).

3.3.2 Exclusion Criteria

A. Participants who have received any systemic anti-cancer treatment for SCLC, including surgical treatment, local radiotherapy, cytotoxic drug treatment, targeted drug treatment and experimental treatment;
B. Administration of any Chinese medicine against cancer before administration of the drug;
C. Participants with cancer other than SCLC (excluding cervical carcinoma in situ, cured basal cell carcinoma, bladder epithelial tumor [including TA and tis]) within five years before the start of this study;
D. Participants with any unstable systemic disease (including active infection, uncontrolled hypertension), unstable angina pectoris, angina pectoris starting in the last three months, congestive heart failure (>= NYHA) Grade II), myocardial infarction (6 months before admission), severe arrhythmia requiring drug treatment, liver, kidney or metabolic diseases;
E. With activate or suspectable autoimmune disease, or autoimmune paracancer syndrome requiring systemic treatment;
F. Antibiotics were used to treat the infection for 4 weeks prior to the start of the trial;

G. Participants who were systemically treated with corticosteroids (prednisone or other corticosteroids >10 mg/ day) or other immunosuppressive agents within 2 weeks prior to first administration. In the absence of active autoimmune disease, inhaled or topical corticosteroids and adrenal hormone replacement therapy with a dose of less than 10 mg/ day of prednisone are permitted;

H. Participants who are allergic to the test drug or any auxiliary materials

I. Participants with active hepatitis B, hepatitis C or HIV;

J. The vaccine was administered within 4 weeks of the start of the trial;

K. Participants who have undergone major surgery or severe trauma in other systems within 2 months before the start of this trial;

L. Pleural effusion, pericardial effusion or ascites that are not clinically controlled and require pleural puncture or abdominal puncture drainage within 2 weeks before inclusion;

M. The patients have active pia meningioma, uncontrolled or untreated brain metastases.

N. Pregnant or lactating women;

O. Participants suffering from nervous system diseases or mental diseases that cannot cooperate;

P. Participated in another therapeutic clinical study.

Q. Other factors that researchers think it is not suitable for enrollment.

3.4 Estimated research duration

According to the enrollment of 2 cases per month, it will take about 20 months to finish enrollment. It is expected to be completed in October 2022.

4. Treatment

4.1 Treatment Protocol

4.1.1 Induction Therapy

Patients will receive a 4-cycle induction therapy, with each cycle lasting 3 weeks. The treatment regimen for each cycle is as follows:

TQB2450 Injection: 1200mg per dose, administered intravenously on the first day of each cycle.

Platinum + Etoposide: Either cisplatin 75 mg/m² or carboplatin AUC 5, combined with etoposide 100 mg/m², administered intravenously. Platinum-based chemotherapy is given on the first day of each cycle, and etoposide is administered on days 1, 2, and 3 of each cycle.

4.1.2 Surgery or Radiotherapy

After the induction therapy, patients with LS-SCLC will undergo either surgery (for eligible curative surgery candidates) or radiotherapy, based on multidisciplinary discussions and patient preferences.

4.1.3 Maintenance Therapy

Two cycles of TQB2450 + platinum + etoposide will be administered. Subsequently, TQB2450 will continue for up to one year with each cycle lasting 3 weeks. Treatment will continue until any of the following conditions occur:

- Intolerable toxicity.
- Disease progression.
- Investigator deems no further clinical benefit.
- Withdrawal of informed consent.
- Initiation of new treatment (excluding radiotherapy).
- Death.

4.2 Preparation and Storage

4.2.1 TQB2450

TQB2450 is an innovative anti-PD-L1 fully humanized monoclonal antibody with a novel sequence. It falls under the category of innovative monoclonal antibodies, classified as Therapeutic Biologics Class 1. It is a monoclonal antibody that can bind to PD-L1, blocking the interaction between PD-L1 and PD-1/B7.1 receptors.

Preparation and Administration:

Before administration, inspect the drug for the presence of particulate matter and discoloration. The drug is a clear to slightly opalescent, colorless to pale yellow liquid. If the solution appears cloudy, discolored, or contains visible particles, it should be discarded. Do not shake the vial.

Prepare the infusion solution as follows:

Withdraw 20 mL of anti-PD-L1 fully humanized monoclonal antibody injection from the vial.

Dilute in a polyvinyl chloride (PVC), polyethylene (PE), or polyolefin (PO) infusion bag with 0.9% sodium chloride injection to a total volume of 250 mL.

Dilute only with 0.9% sodium chloride injection.

Mix the diluted solution by gently inverting; do not shake.

Discard any partially used or empty vials.

Storage of Infusion Solution:

TQB2450 does not contain preservatives. Once prepared, administer immediately. If the diluted infusion solution is not used immediately, store as follows:

At room temperature for up to 6 hours, including storage and infusion time.

In a refrigerator at 2°C–8°C for no more than 24 hours. Do not freeze. Do not shake.

Administration:

For the initial administration, use an infusion set equipped with a sterile, non-pyrogenic, low-protein adsorption in-line filter (pore size: 0.2–0.22μm), completing the 60-minute infusion. If the initial infusion is tolerated, subsequent infusions should be completed in over 30 minutes. Do not share the infusion set with other drugs.

4.2.2 Chemotherapy Drugs

The management (handling, storage, administration, and disposal) of platinum-based chemotherapy and etoposide will follow the drug's summary of product characteristics (SmPC). For further details, refer to the prescribing information from the respective chemotherapy drug manufacturers.

4.3 Dosage, Administration, and Compliance

The dosing schedule for TQB2450 and chemotherapy is outlined in the table below. Adverse events (AEs) in all patients will be continuously monitored. Treatment adjustments, such as dose delays, dose reductions, suspension of administration, or termination of administration, will be made based on specific laboratory test results and AE criteria.

Treatment Selection and Administration Schedule				
Administration Sequence	Drug	Dosage/Route	Induction Therapy[a]	Maintenance Therapy
1	TQB2450	1200 mg (IV)	Day 1 of each cycle:<br>Cycle 1: Infusion for 60 minutes (pre-chemotherapy waiting ≥60 minutes)<br>Cycles 2-4: Infusion for 30-60 minutes (pre-chemotherapy waiting ≥60 minutes for Cycle 2, subsequently ≥30 minutes)	Day 1 of each cycle: Infusion for 30-60 minutes
2	Cisplatin or	75 mg/m^2 (IV)	Day 1 of each cycle:	Terminate

	Carboplatin	AUC 5 (IV)	Infusion for over 2 hours	treatment
3	Etoposide	100 mg/m^2 (IV)	Day 1 to Day 3 of each cycle: Infusion for over 60 minutes	Terminate treatment

Abbreviation: IV = Intravenous

a If the initial infusion is tolerated, subsequent infusions should be completed in over 30 minutes. Intravenous push or rapid injection is not permissible.

4.4 Drug Dose Adjustment

4.4.1 TQB2450 Dose Adjustment

It is not recommended to reduce the drug dosage.

- **Discontinue TQB2450 in the presence of any of the following, TQB2450 may be resumed when the patient's adverse reactions recover to Grade 0–1:**

Grade 2 pneumonia

Aspartate transaminase (AST) or alanine transaminase (ALT) greater than 3 and up to 5 times the upper limit of normal (ULN) or total bilirubin greater than 1.5 and up to 3 times ULN

Grade 2 or 3 diarrhea or colitis

Symptomatic hypophysitis, adrenal insufficiency, hypothyroidism, hyperthyroidism, or Grade 3 or 4 hyperglycemia

Grade 2 ocular inflammation toxicity

Grade 2 or 3 pancreatitis or amylase/lipase levels increased to Grade 3 or 4 (greater than 2.0 times ULN)

Grade 3 or 4 infection

Grade 2 infusion-related reaction

Grade 3 rash

- **Immediately terminate TQB2450 in the presence of:**

Grade 3 or 4 pneumonia

Grade 4 diarrhea or colitis

Grade 4 hypophysitis

Myasthenic syndrome/myasthenia gravis, Guillain-Barré, or aseptic meningitis (any grade)

Grade 3 or 4 ocular inflammation toxicity

Grade 4 or any recurrent pancreatitis

Grade 3 or 4 infusion-related reaction

Grade 4 rash

4.4.2 Chemotherapy Dose Adjustment

Chemotherapy dose adjustment should be based on the investigator's clinical judgment, following prescription information, and clinical practice guidelines. The required dose of chemotherapy is calculated based on baseline body weight. Dose adjustment is required if the patient's weight changes by ≥10% from baseline (or new reference weight). If the weight change is <10%, chemotherapy dose adjustment is not necessary.

Toxicities related to the study drug must resolve to baseline, Grade 0, or Grade 1 before the next dose, except for alopecia or Grade 2 fatigue. Each chemotherapy drug allows a maximum of two dose reductions. Once the dose is reduced, all subsequent administrations should be maintained at the reduced dose or further reduced if necessary. There is no dose escalation in this study. If another dose reduction is needed, chemotherapy drug treatment must be terminated.

In the case of chemotherapy-related toxicity, if adverse events (AE) resolve within 10 days, chemotherapy is administered. Based on the scheduled chemotherapy date, chemotherapy or TQB2450 administration will be resynchronized in the subsequent cycle. If AE does not resolve within 10 days, chemotherapy is not administered. If AE resolves within 21 days, chemotherapy or TQB2450 is administered on the first day of the next treatment cycle according to the original schedule.

If chemotherapy is delayed due to toxicity/adverse events, it can be delayed for up to 21 days. All subsequent chemotherapy administrations must be rescheduled based on the last chemotherapy administration date.

Preventive measures:

Neutropenia: Febrile or other signs of infection must be promptly evaluated, and active treatment should follow clinical practice guidelines and/or guidance.

Nephrotoxicity:

Nephrotoxicity is a common adverse event of cisplatin. Oral hydration is recommended. Avoid

the use of nephrotoxic drugs such as aminoglycoside antibiotics.

Patients with a creatinine clearance (CrCl) < 60 mL/min should not receive cisplatin treatment.

4.5 Criteria for Terminating Chemotherapy

Except for specific circumstances mentioned above, chemotherapy drugs should be discontinued if any of the following occur:

Any Grade 4 peripheral neuropathy requires discontinuation of cisplatin.

Persistent Grade 3 sensory abnormalities require discontinuation of cisplatin.

Grade 3 or 4 drug-related thrombocytopenia with clinically significant bleeding.

Grade 3 or 4 total bilirubin elevation and Grade 4 ALT or AST elevation require discontinuation of etoposide.

Discontinuation is needed in case of any drug-related liver function test abnormality meeting the following criteria:

AST or ALT >5–10×ULN, duration >2 weeks.

ALT or AST >10×ULN.

Total bilirubin >5×ULN.

Simultaneous occurrence of AST or ALT >3×ULN and total bilirubin >2×ULN.

Discontinue cisplatin administration when creatinine clearance related to cisplatin decreases to <30 mL/min (calculated using Cockcroft-Gault formula).

Discontinue etoposide administration when creatinine clearance decreases to <15 mL/min.

Terminate cisplatin or etoposide administration if the same drug-related adverse event recurs after two dose reductions.

Discontinue the drug for any Grade 3 or 4 drug-related hypersensitivity or infusion reaction. Drugs considered unrelated to hypersensitivity or infusion reaction can continue to be administered.

Any Grade 4 AE deemed related to the study drug and not suitable for management by dose reduction requires discontinuation. Drugs considered unrelated to the event can continue to be administered.

If toxicity does not resolve within 21 days, the administration of that drug component will be terminated.

For toxicities not listed above, investigators should make medical judgments based on the

patient's health status and clinical standards to determine whether chemotherapy should be terminated.

5. Combination Therapy

5.1 Permitted Combination Drugs/Treatments

The use of most concomitant drugs and treatments deemed necessary by the investigator and in line with clinical treatment standards, for supportive care (e.g., antiemetics, antidiarrheals), and in the best interest of the patient is allowed.

Before the next administration, systemic corticosteroids required to control immune-related adverse events (irAEs) must be gradually tapered (see Appendix 5) and maintained at non-immunosuppressive levels (prednisone or equivalent ≤10 mg/day). Short-term use of steroids for prophylactic treatment (e.g., in patients allergic to contrast agents used in imaging diagnosis) is allowed.

The use of hematopoietic growth factors (i.e., G-CSF or GM-CSF) to treat febrile neutropenia is allowed. Any growth factor support must be documented in the patient's records and case report form (CRF).

During maintenance treatment, patients are allowed prophylactic cranial irradiation (PCI) according to clinical standards.

After achieving CR or PR in any group after Cycle 4, subjects may be given PCI at the discretion of the treating investigator. Brain imaging must be performed before PCI. Administration of the study drug can continue during PCI; however, if it is necessary to interrupt study treatment, it must be resumed within 2 weeks after completing PCI. Steroids may be given during and after PCI if needed.

Patients are allowed to use opioid and other drugs for palliative care as needed. Patients must inform the investigator of all concomitant medications used during the study.

Additionally, palliative radiation or other focal ablative treatments for non-target lesion areas are allowed if clinically necessary based on the investigator's judgment and consultation with the medical monitor. Tumor assessment should preferably be conducted before these patients undergo radiation to exclude disease progression. Treatment does not need to be paused during palliative radiation.

5.2 Prohibited or Restricted Combination Drugs/Treatments

During the screening period and TQB2450 administration, the use of the following drugs is prohibited or restricted:

Immunosuppressive agents (except for drugs used to treat drug-related adverse events).

Systemic corticosteroids >10 mg/day (prednisone or equivalent), except for drugs used to treat or control drug-related adverse events (according to the study protocol), or as premedication for chemotherapy, or for short-term prophylactic treatment.

Live vaccines within 28 days before enrollment and 60 days after the last dose of TQB2450.

Herbal medicines with immunostimulatory properties (such as mistletoe extract) or known to potentially interfere with liver or other major organ functions (such as goldthread). Patients must inform the investigator of all herbal medicines used during the study. The use of herbal medicines for cancer treatment is not recommended due to unknown pharmacokinetics, safety profiles, and potential drug-drug interactions. However, patients may use herbal medicines with the investigator's agreement, provided there are no known interactions with any study treatments. As mentioned earlier, herbal treatments for cancer are prohibited.

During the screening period and any administration of study treatments, the use of the following drugs is prohibited or restricted:

Concurrent any other antitumor therapy (i.e., other chemotherapy, hormonal therapy, immunotherapy, or standard/experimental drugs for cancer treatment [including herbal medicines and traditional Chinese medicines]).

Extensive radiotherapy (except for local, palliative radiotherapy that does not affect the assessment of target lesion tumors).

6. Research Assessment and Procedures

The planned research assessment table is available in Appendix 1. Throughout the entire study, patient safety and tolerance will be closely monitored. Every patient must undergo all assessments, and the information should be documented in the patient's medical records. Administration of the study drug is only permitted after reviewing clinical assessments and laboratory test values (obtained before any drug administration) and deeming them acceptable according to the protocol guidelines.

6.1 Screening

Screening assessments will be conducted within 28±7 days before enrollment. Patients agreeing to participate will sign an informed consent form before commencing the screening procedures. The screening period commences on the first day of screening procedures. All patients undergo a physical examination, laboratory assessments, and pulmonary function tests (including spirometry and oxygenation assessment) at baseline (see Appendix 1). Screening assessments may be repeated as necessary; the investigator will evaluate patient eligibility based on the most recent screening assessment results.

6.1.1 Informed Consent and Screening

A voluntary, written informed consent form must be obtained before any study-specific procedures are conducted. All informed consent forms for enrolled and screening-failed patients will be retained at the study center. All screening assessments must be completed and reviewed, and patients can only be enrolled after confirming that they meet all eligibility criteria.

6.1.2 Patient Identification

Upon obtaining the informed consent form, a unique patient identification number will be assigned to potential study participants.

6.1.3 Demographic Data and Medical History

Demographic data include birth year (or age) and gender.

Medical history includes any clinically significant history of diseases, surgical history, or cancer history; reproductive status (i.e., fertility or infertility); and alcohol and smoking history (i.e., past, present, or never).

Cancer history involves an assessment of past surgeries, radiotherapy, and medication treatments, including start and stop dates, best response, and reasons for discontinuation. Imaging results conducted before study enrollment may be collected for the investigator's review.

6.1.4 Fertile Women and Contraception

Fertile women are those with the physiological ability to become pregnant. Refer to Appendix 7 for guidelines on contraception and the definitions of "fertile women" and "infertile women."

6.2 Confirmation of Eligibility for Enrollment

The investigator will assess and confirm the eligibility of each patient. Before confirming

eligibility, all screening procedure results and relevant medical history must be obtained. All inclusion criteria must be met, and no exclusion criteria should be present.

6.3 Assessment of safety

6.3.1 Vital Signs

Vital signs include body temperature (°C), respiratory rate, heart rate, and blood pressure (systolic and diastolic) measured after the patient has rested for 10 minutes while in a seated position. Height and weight should be measured and recorded in the Case Report Form (CRF) only at baseline. Vital signs need to be assessed at the beginning of each cycle during the screening period (-D28 - D0), induction and maintenance periods before the start of each medication, post-treatment evaluation periods, and safety assessment periods. Additionally, measurements may be taken during infusion and 30 minutes after infusion based on clinical need.

6.3.2 Physical Examination

A comprehensive physical examination, including assessment of the head, eyes, ears, nose, throat, cardiovascular system, skin, musculoskeletal system, respiratory system, gastrointestinal tract, and nervous system, will be conducted at the beginning of each cycle during the screening period (-D28 - D0), induction and maintenance periods before the start of each medication, post-treatment evaluation periods, and safety assessment periods. Any abnormalities discovered during the screening period will be graded according to NCI-CTCAE v5.0, and appropriate disease/condition terminology will be recorded in the CRF.

6.3.3 ECOG Performance Status Score

ECOG Performance Status (PS) will be assessed according to the ECOG PS scoring criteria at the beginning of each cycle during the screening period (-D28 - D0), induction and maintenance therapy periods on the first day of each cycle, post-treatment evaluation periods, and safety visit periods.

6.3.4 Laboratory Tests

Laboratory tests, including serum biochemistry, hematology, coagulation, and urine analysis, will be conducted, and specific information will be collected as follows. Hematological and serum biochemistry tests will be performed at the beginning of each cycle during the screening period, induction and maintenance therapy periods before the start of each medication, post-treatment

evaluation periods, and safety assessment periods. The tests include hepatitis detection (HBsAg, anti-HBs, HBeAg, anti-HBe, anti-HBc, and HBcAb IgM), HCV antibody testing, HIV antibody testing, thyroid function (FT3, FT4, TSH, and triiodothyronine), complete blood count (hemoglobin, red blood cell count, white blood cell count, neutrophil count, lymphocyte count, and platelet count), serum biochemistry (LDH, ALP, GTP, TC, TG, TP, ALB, and GLU), liver and kidney function, and electrolytes. Additionally, cardiac enzyme spectrum (CK, CK-MB, Mb, cTnI), coagulation function tests (PT, APTT, INR, TT, FDP), and urinalysis (protein, glucose, occult blood) will be conducted.

6.3.5 Pulmonary Function Test

Pulmonary function tests, including vital capacity, oxygenation assessment, and pulse oxygen saturation at rest, will be conducted at the screening period (-D28 - D0), before surgery/radiotherapy, post-treatment evaluation periods, and safety visit periods. These tests will help determine the patient's suitability for participation in the study. Patients with significantly impaired lung function.

6.3.6 Electrocardiogram (ECG)

ECG recordings will be obtained during the screening period, post-treatment evaluation periods, safety follow-up visits, and when clinically indicated. For safety monitoring purposes, the investigator must review and sign all ECG scans, indicating the date. The paper or electronic version of the ECG tracing will be preserved as a part of the patient's permanent study record at the research center.

6.3.7 Radiological Test and Assessment

ET-CT and chest CT imaging were performed within 28 days before the first cycle of treatment to determine the TNM stage. Endobronchial ultrasound was performed to diagnose the pathological stage of lymph node. Additional chest CT imaging was performed 3-4 weeks after the completion of each 2 cycle. Radiologic response was assessed according to Response Evaluation Criteria in Solid Tumors version 1.1. Tumor assessments will continue until radiological disease progression.

Pathological examination was performed by two independent pathologists. PD-L1 expression was measured by 22C3 pharmDx kit (Dako, Carpinteria, CA, USA). Primary tumors were assessed for the percentage of residual viable tumor (RVT) on routine hematoxylin and eosin staining after operation. Tumors with no more than 10% RVT were considered as major pathological response (MPR), and tumors and lymph nodes without RVT were considered as pathological complete

response (PCR).

6.4 Biomarker

Archived tumors for biomarker evaluation will require dedicated personnel responsible for transportation, storage, and handling.

Tumor tissue specimens, in addition to measuring PD-L1 expression, may also be used to evaluate exploratory, predictive biomarkers related to effectiveness or clinical benefit of TQB2450, such as tumor mutation load (TMB) and immune-related gene expression profile (GEP), transcriptome sequencing, etc.

For patients whose disease progression is confirmed during the study, selective biopsies can be performed at accessible tumor sites to explore resistance mechanisms. If feasible, follow-up biopsy samples should preferably be taken from the same tumor lesion as the baseline biopsy.

6.5 Follow-up Period

Following the completion of treatment, the study enters the follow-up period. Follow-up assessments include monitoring symptoms, chest CT scans, whole-body ECT in case of bone pain, and head MRI in case of headaches, vomiting, or neurological symptoms such as asymmetrical limbs or unsteady gait. These follow-up assessments occur every 3 months ±14 days, continuously for 2 years. If, after 2 years from the end of treatment, there is no recurrence or metastasis, the study transitions to the telephone survival follow-up phase, with phone calls every 3 months ±14 days. The content of these telephone follow-ups includes assessing survival status, recurrence time, recording the date and cause of death if applicable, and documenting any subsequent anticancer treatments after progression. In the event of recurrence or metastasis, the study progresses to the post-progression survival follow-up phase, with telephone follow-ups every 3 months ±14 days. These follow-ups encompass survival status, recording the date and cause of death if applicable, and documenting any subsequent anticancer treatments after progression.

6.6 Visit Windows

All visits are conducted within ±3 days of the planned date unless otherwise specified (see Appendix 1). Unless an acceptable window is specified, all assessments will be performed on the designated visit date. If the scheduled study visit coincides with holidays, weekends, or other events, the visit should be scheduled on the nearest feasible date (refer to the visit window in Appendix 1),

and subsequent visits should follow the planned schedule, starting from the first day of the first cycle, occurring every 3 weeks.

7. Statistical methods and sample size

7.1 Statistical Analysis

7.1.1 Analysis Sets

The Intention-to-Treat (ITT) analysis set comprises all enrolled patients and serves as the primary analysis set for all effectiveness analyses, including Event-Free Survival (EFS) and Overall Survival (OS).

The Safety Analysis Set includes all patients who received at least one dose of any study drug and will be used for safety analysis. Analysis will be conducted based on the actual treatment received by each patient.

7.1.2 Demographics and Baseline Characteristics

Demographics and other baseline characteristics of the ITT analysis set will be summarized using descriptive statistical methods. Continuous variables include age, weight, time since initial LS-SCLC diagnosis, and categorical variables include histology, prior systemic therapy, prior radiation, disease stage, PD-L1 expression, gender, ECOG PS, smoking status, and metastatic sites.

7.2 Safety Analysis

Safety will be assessed by monitoring and recording all Serious Adverse Events (SAE) graded according to NCI-CTCAE 5.0. Safety analysis will also include descriptive statistical analysis of all safety data in the safety analysis set. The frequency of SAEs occurring within 90 days after the last TQB2450 administration or within 30 days after starting new anticancer therapy (beyond the scope of this study regimen) or surgery will be reported (whichever occurs first).

7.3 Effectiveness Analysis

(1) Objective Response Rate (ORR)

ORR is the proportion of patients with Complete Response (CR) or Partial Response (PR) according to RECIST v1.1 among all enrolled patients with measurable lesions at baseline. Patients without post-baseline assessment will be considered non-responders.

(2) Event-Free Survival (EFS)

EFS is defined as the time from enrollment to the first documented disease progression,

recurrence (evaluated by RECIST v1.1), or death from any cause (whichever occurs first). EFS analysis will be conducted in the ITT analysis set. Kaplan-Meier methods will be used to estimate median EFS and plot Kaplan-Meier curves. Patients without disease progression, recurrence, or death at the time of analysis will be censored at their last valid tumor assessment date.

(3) Overall Survival (OS)

OS is defined as the time from enrollment to death from any cause. OS analysis will be conducted in the ITT analysis set. Kaplan-Meier methods will be used to estimate median OS and plot Kaplan-Meier curves. Data from patients who have not died at the time of analysis will be censored at the last known survival date.

(4) Major Pathological Response Rate (MPR)

MPR is defined as the proportion of patients with a major pathological response among those who underwent surgical pathological assessment. A major pathological response is defined as residual tumor cells occupying ≤10% of the resected tumor.

(5) Complete Pathological Response Rate (PCR)

PCR is defined as the proportion of patients with a complete pathological response among those who underwent surgical pathological assessment. A complete pathological response is defined as no visible residual tumor cells in both tumor and lymph nodes.

(6) Health-Related Quality of Life (HRQoL)

Mean changes from baseline in scores (along with 95% CI using the normal approximation) will be assessed. The proportion of patients with clinically significant changes at each assessment time point will be calculated. Analysis will be conducted only for patients who underwent both baseline and at least one post-baseline assessment. Summarization will be performed in the ITT analysis set.

7.4 Exploratory Analysis

The distribution of PD-L1 expression in the ITT analysis set will be examined. Any potential associations between PD-L1 expression and treatment outcomes (PFS, OS, ORR) will be explored. Other potential predictive biomarkers, including but not limited to PD-L1 expression, gene expression profile (GEP), tumor mutation burden (TMB), and tumor immune microenvironment cells, may be evaluated before and/or at the time of surgery /disease progression.

7.5 Sample Size Consideration

This is an exploratory trial, considering lack of information on the effectiveness of surgery in patients with LS-SCLC, this study aims to enroll 40 patients.

8. Reference

1. Siegel, R.L., K.D. Miller, and A. Jemal, Cancer statistics, 2019. CA Cancer J Clin, 2019. 69(1): p. 7-34.
2. 陈万青，张思维，郑荣寿，雷正龙，李光琳，邹小农，and 赵平，中国肿瘤登记地区 2007 年肿瘤发病和死亡分析. 中国肿瘤, 2011. 20(3): p. 162-169.
3. Chen, W., R. Zheng, P.D. Baade, et al, Cancer statistics in China, 2015. CA Cancer J Clin, 2016. 66(2): p. 115-132.
4. 段纪俊，严亚琼，杨念念，曾晶，郑荣寿，张思维，and 陈万青，中国恶性肿瘤发病与死亡的国际比较分析. 中国医学前沿杂志（电子版）, 2016. 8(7): p. 17-23.
5. Kalemkerian, G.P. and B.J. Schneider, Advances in Small Cell Lung Cancer. Hematol Oncol Clin North Am, 2017. 31(1): p. 143-156.
6. National Comprehensive Cancer Network (NCCN) Guidelines for Small Cell Lung Cancer 2019. Version 1
7. Schmid, S. and M. Fruh, Immune checkpoint inhibitors and small cell lung cancer: what's new? J Thorac Dis, 2018. 10(Suppl 13): p. S1503-s1508.
8. Pesch, B., B. Kendzia, P. Gustavsson, K.H. et al, Cigarette smoking and lung cancer--relative risk estimates for the major histological types from a pooled analysis of case-control studies. Int J Cancer, 2012. 131(5): p. 1210-1219.
9. Varghese, A.M., M.F. Zakowski, et al, Small-cell lung cancers in patients who never smoked cigarettes. J Thorac Oncol, 2014. 9(6): p. 892-896.
10. Evans, W.K., F.A. Shepherd, R. Feld, D. Osoba, P. Dang, and G. Deboer, VP-16 and cisplatin as first-line therapy for small-cell lung cancer. J Clin Oncol, 1985. 3(11): p. 1471-1477.
11. Einhorn, L.H., J. Crawford, et al, Cisplatin plus etoposide consolidation following cyclophosphamide, doxorubicin, and vincristine in limited small-cell lung cancer. J Clin Oncol, 1988. 6(3): p. 451-456.
12. Sundstrom, S., R.M. Bremnes, S. Kaasa, et al, Cisplatin and etoposide regimen is superior to cyclophosphamide, epirubicin, and vincristine regimen in small-cell lung cancer: results from a randomized phase III trial with 5 years' follow-up. J Clin Oncol, 2002. 20(24): p. 4665-4672.
13. Warde, P. and D. Payne, Does thoracic irradiation improve survival and local control in limited-stage small-cell carcinoma of the lung? A meta-analysis. J Clin Oncol, 1992. 10(6): p. 890-895.
14. Fox, W. and J.G. Scadding, Medical Research Council comparative trial of surgery and radiotherapy for primary treatment of small-celled or oat-celled carcinoma of bronchus. Ten-year follow-up. Lancet, 1973. 2(7820): p. 63-65.
15. Lad, T., S. Piantadosi, P. Thomas, D. Payne, J. Ruckdeschel, and G. Giaccone, A prospective randomized trial to determine the benefit of surgical resection of residual disease following response of small cell lung cancer to combination chemotherapy. Chest, 1994. 106(6 Suppl): p. 320s-323s.
16. Schreiber, D., J. Rineer, J. Weedon, D. Vongtama, A. Wortham, A. Kim, P. Han, K. Choi, and M. Rotman, Survival outcomes with the use of surgery in limited-stage small cell lung cancer: should its role be re-evaluated? Cancer, 2010. 116(5): p. 1350-1357.
17. Luchtenborg, M., S.P. Riaz, E. Lim, et al, Survival of patients with small cell lung cancer undergoing lung resection in England, 1998-2009. Thorax, 2014. 69(3): p. 269-273.
18. Yu, J.B., R.H. Decker, F.C. Detterbeck, and L.D. Wilson, Surveillance epidemiology and end results evaluation

of the role of surgery for stage I small cell lung cancer. J Thorac Oncol, 2010. 5(2): p. 215-219.

19. Weksler, B., K.S. Nason, M. Shende, et al, Surgical resection should be considered for stage I and II small cell carcinoma of the lung. Ann Thorac Surg, 2012. 94(3): p. 889-893.

20. Yang, C.F., D.Y. Chan, P.J. Speicher, et al, Role of Adjuvant Therapy in a Population-Based Cohort of Patients With Early-Stage Small-Cell Lung Cancer. J Clin Oncol, 2016. 34(10): p. 1057-1064.

21. Tsuchiya, R., K. Suzuki, Y. Ichinose, et al, Phase II trial of postoperative adjuvant cisplatin and etoposide in patients with completely resected stage I-IIIa small cell lung cancer: the Japan Clinical Oncology Lung Cancer Study Group Trial (JCOG9101). J Thorac Cardiovasc Surg, 2005. 129(5): p. 977-983.

22. Yang, C.J., D.Y. Chan, S.A. Shah, B.A. Yerokun, X.F. Wang, T.A. D'Amico, M.F. Berry, and D.H. Harpole, Jr., Long-term Survival After Surgery Compared With Concurrent Chemoradiation for Node-negative Small Cell Lung Cancer. Ann Surg, 2018. 268(6): p. 1105-1112.

23. Fujimori, K., A. Yokoyama, Y. Kurita, and M. Terashima, A pilot phase 2 study of surgery after induction chemotherapy for resectable stage I to IIIA small cell lung cancer. Chest, 1997. 111(4): p. 1089-1093.

24. Veronesi, G., P. Scanagatta, F. Leo, et al, Adjuvant Surgery after Carboplatin and VP16 in Resectable Small Cell Lung Cancer. Journal of Thoracic Oncology, 2007. 2(2): p. 131-134.

25. Fruh M, De Ruysscher D, Popat S, et al. Small-cell lung cancer (SCLC): ESMO clinical practice guidelines for diagnosis, treatment and follow-up. Ann Oncol 2013;24(Suppl 6):vi99-105.

26. Evans WK, Shepherd FA, Feld R, et al. Vp-16 and cisplatin as first-line therapy for small-cell lung cancer. J Clin Oncol 1985;3:1471-7.

27. Skarlos DV, Samantas E, Kosmidis P, et al. Randomized comparison of etoposide-cisplatin vs. etoposide-carboplatin and irradiation in small cell lung cancer. A Hellenic co-operative oncology group study. Ann Oncol 1994;5:601-7.

28. Okamoto H, Watanabe K, Kunikane H, et al. Randomised phase III trial of carboplatin plus etoposide vs split doses of cisplatin plus etoposide in elderly or poor-risk patients with extensive disease small-cell lung cancer. JCOG 9702. Br J Cancer 2007;97:162-9.

29. Rossi A, Di Maio M, Chiodini P, et al. Carboplatin- or cisplatin-based chemotherapy in first-line treatment of small cell lung cancer: the COCIS meta-analysis of individual patient data. J Clin Oncol 2012;30:1692-8.

30. Roth BJ, Johnson DH, Einhorn LH, et al. Randomized study of cyclophosphamide, doxorubicin, and vincristine versus etoposide and cisplatin versus alternation of these two regimens in extensive small cell lung cancer: a phase III trial of the Southeastern Cancer Study Group. J Clin Oncol 1992;10:282-91.

31. Pujol JL, Daures JP, Riviere A, et al. Etoposide plus cisplatin with or without the combination of 4'-epidoxorubicin plus cyclophosphamide in treatment of extensive small cell lung cancer: a French Federation of Cancer Institutes multicenter phase III randomized study. J Natl Cancer Inst 2001;93:300-8.

32. Noda K, Nishiwaki Y, Kawahara M, et al. Irinotecan plus cisplatin compared with etoposide plus cisplatin for extensive small cell lung cancer. N Engl J Med 2002;346:85-91.

33. Eckardt JR, von Pawel J, Papai Z, et al. Open-label, multicenter, randomized, phase III study comparing oral topotecan/cisplatin versus etoposide/cisplatin as treatment for chemotherapy-naive patients with extensive-disease small-cell lung cancer. J Clin Oncol 2006;24:2044-51.

34. Hanna N, Bunn PA Jr, Langer C, et al. Randomized phase III trial comparing irinotecan/cisplatin with etoposide/cisplatin in patients with previously untreated extensive-stage disease small cell lung cancer. J Clin Oncol 2006;24:2038-43.

35. Rudin CM, Salgia R, Wang X, et al. Randomized phase II study of carboplatin and etoposide with or without the bcl-2 antisense oligonucleotide oblimersen for extensive-stage small cell lung cancer: CALGB 30103. J Clin Oncol 2008;26:870-6.

36. Socinski MA, Smit EF, Lorigan P, et al. Phase III study of pemetrexed plus carboplatin compared with etoposide plus carboplatin in chemotherapy-naive patients with extensive-stage small cell lung cancer. J Clin Oncol 2009;27:4787-92.

37. Nagel S, Kellner O, Engel-Riedel W, et al. Addition of darbepoetin alfa to dose-dense chemotherapy: results from a randomized phase II trial in small-cell lung cancer patients receiving carboplatin plus etoposide. Clin Lung Cancer 2011;12:62-9.

38. Schmittel A, Sebastian M, Fischer von Weikersthal L, et al. A German multicenter, randomized phase III trial comparing irinotecan-carboplatin with etoposidecarboplatin as first-line therapy for extensive-disease small-cell lung cancer. Ann Oncol 2011;22:1798-804.

39. Gong, J., A. Chehrazi-Raffle, S. Reddi, and R. Salgia, Development of PD-1 and PD-L1 inhibitors as a form of cancer immunotherapy: a comprehensive review of registration trials and future considerations. J Immunother Cancer, 2018. 6(1): p. 8.

40. Yoshimura, A., T. Yamada, A. Miyagawa-Hayashino, Y. et al, Comparing three different anti-PD-L1 antibodies for immunohistochemical evaluation of small cell lung cancer. Lung Cancer, 2019. 137: p. 108-112.

41. Azuma, T., S. Yao, G. Zhu, A.S. Flies, S.J. Flies, and L. Chen, B7-H1 is a ubiquitous antiapoptotic receptor on cancer cells. Blood, 2008. 111(7): p. 3635-3643.

42. Hirano, F., K. Kaneko, H. Tamura, H. et al. Chen, Blockade of B7-H1 and PD-1 by monoclonal antibodies potentiates cancer therapeutic immunity. Cancer Res, 2005. 65(3): p. 1089-1096.

43. Paz-Ares, L., M. Dvorkin, Y. Chen, N. et al, Durvalumab plus platinum-etoposide versus platinum-etoposide in first-line treatment of extensive-stage small-cell lung cancer (CASPIAN): a randomised, controlled, open-label, phase 3 trial. Lancet, 2019. 394(10212): p. 1929-1939.

44. Horn, L., A.S. Mansfield, A. Szczesna, et al, First-Line Atezolizumab plus Chemotherapy in Extensive-Stage Small-Cell Lung Cancer. N Engl J Med, 2018. 379(23): p. 2220-2229.

45. Soar J, Pumphrey R, Cant A, et al. Emergency treatment of anaphylactic reactions--guidelines for healthcare providers. Resuscitation. 2008;77(2):157-69.

46. Haanen JBAG, Carbonnel F, Robert C, et al. Management of toxicities from immunotherapy: ESMO Clinical Practice Guidelines for diagnosis, treatment, and follow-up. Ann Oncol. 2017:28 (suppl 4):iv119-iv142.

47. Brahmer JR, Lacchetti C, Schneider BJ, et al. Management of immune-related adverse events in patients treated with immune checkpoint inhibitor therapy: American Society of Clinical Oncology Clinical Practice Guideline. J Clin Oncol. 2018.

48. Go SI, Keam B, Kim TM, et al. Clinical significance of downstaging in patients with limited-disease small-cell lung cancer. Clin Lung Cancer. 2014;15(2):e1-e6.

49. Noda K, Nishiwaki Y, Kawahara M, et al. Irinotecan plus cisplatin compared with etoposide plus cisplatin for extensive small-cell lung cancer. N Engl J Med. 2002;346(2):85-91.

50. Spigel DR, Townley PM, Waterhouse DM, et al. Randomized phase II study of bevacizumab in combination with chemotherapy in previously untreated extensive-stage small-cell lung cancer: results from the SALUTE trial. J Clin Oncol. 2011;29(16):2215-2222.

Appendix 1. Evaluation flow sheet

Evaluation Items	Screening [1]		Induction Phase (every 3 weeks, 4 cycles)			Surgery/ Radiotherapy[10]	Maintenance Therapy (every 3 weeks)	Safety Visit [2]	Survival Follow-up [3]
Time Windows (Days)	-28 - 0 days	-7 - 0 days	1(±3) days	8(±2) days	21(±2) days	-	1(±3) days	30±7days	every 3 months ± 14 days.
Informed Consent	X								
Inclusion/Exclusion Criteria	X								
Demographic Information/Medical History	X								
Vital Signs/Height and Weight	X		X				X	X	
Physical Examination	X		X				X	X	
ECOG Performance Status	X		X				X	X	
12-Lead ECG	X						According to Clinical Needs		
Adverse Events [4]	X		X	X[9]	X[9]		X	X	X
Concomitant Medications and Therapies	X		X	X[9]	X[9]		X	X	
Hematology		X		X	X		X	X	
Serum Biochemistry		X			X		X	X	
Liver/Kidney Function and Electrolytes		X		X	X		X	X	
Coagulation Function		X			X		X	X	

Cardiac Enzymes		X			X		X	X	
Thyroid Function [5]	X				X[6]		X[6]	X	
Urinalysis		X			X		According to Clinical Needs	X	
Hepatitis Marker Testing	X						According to Clinical Needs		
HIV Testing	X								
Pregnancy Test [6]		X							
Pulmonary Function Test	X								
Tumor Assessment [7]	X				X		X		
TQB2450 Administration			X				X		
Platinum Drug + Etoposide Administration			X						
Surgery						X			
Pathology Results						X			
Quality of Life Scores [8]	X				X		X	X	
Survival Status									X

Note:

1. Any study-specific tests or procedures can only be conducted after the written informed consent is signed. Standard treatment tests or results conducted within 28 days before obtaining informed consent and during the screening period can be used for the screening assessment without the need for repetition.
2. Safety follow-up visits are required to be conducted within 30 days (±7 days) after the last administration of study treatment or before starting new anticancer treatment, whichever occurs first.
3. After the completion of treatment, a follow-up period will commence, occurring every 3 months ± 14 days for a duration of 2 years. If no relapse or metastasis occurs within 2 years after treatment completion, the study will enter a telephone survival follow-up stage, with telephone follow-ups every 3 months ± 14 days. Follow-up content will include survival status, time of disease recurrence, recording of death date and cause if applicable, and details of any additional anticancer treatments received post progression. Follow-ups will be conducted for all patients

unless they request to withdraw from follow-up.

4. Adverse events (AE) and laboratory abnormalities will be graded according to NCI-CTCAE v5.0. The severity of all adverse events will be assessed. Only serious adverse events (SAE) need to be reported before the initiation of study drug administration for patients who have already signed the informed consent. After the initiation of study drug treatment, all AEs and SAEs, regardless of their relation to the study drug, will be reported until 30 days after the last administration of the study drug (including chemotherapy) or the initiation of new anticancer treatment, whichever occurs first.
5. Thyroid function tests will be conducted during the screening period, every 2 cycles (i.e., on the first day of cycles 2, 4, 6, etc.), and during safety follow-up visits.
6. Pregnancy testing, either urine or serum, must be conducted within 7 days before enrollment for females of childbearing potential, including those who have undergone tubal ligation, with recorded negative results. If urine pregnancy test results are positive or suspicious, a serum pregnancy test must be conducted.
7. If radiological imaging has been performed before obtaining written informed consent, and the timing of the examination is within 28 days before enrollment, these results can be applied, and there is no need for repetition. All measurable and evaluable lesions need to be assessed and recorded at the screening visit. Depending on clinical judgment, the screening period may require a head MRI examination (or a CT scan if MRI is contraindicated or impractical), and if clinically indicated, a bone scan or PET-CT.
8. During the study period, tumor imaging assessments will be conducted approximately every 6 weeks (±7 days) for the first 48 weeks after the first day of the first cycle and then every 9 weeks (±7 days), regardless of treatment dose delays. Patients who discontinue study treatment prematurely for reasons other than disease progression (such as toxicity) will continue tumor assessments as originally planned until the patient starts subsequent anticancer treatment, experiences disease progression, withdraws informed consent, is lost to follow-up, dies, or until the study termination, whichever occurs first.
9. Pulmonary hospital psychological assessments and quality of life assessment scales will be conducted at baseline, every cycle during the induction period, every 2 weeks during the maintenance treatment period, and during safety follow-up visits.
10. On days 8 and 21, AE and concomitant medication reviews can be conducted via telephone.
11. Surgery will only be evaluated in subjects with limited-stage SCLC who are assessed as eligible for curative surgery and are willing to undergo surgery after the completion of the induction phase.
